# BTB-Zinc Finger Oncogenes Are Required for Ras and Notch-Driven Tumorigenesis in *Drosophila*

**DOI:** 10.1371/journal.pone.0132987

**Published:** 2015-07-24

**Authors:** Karen Doggett, Nezaket Turkel, Lee F. Willoughby, Jason Ellul, Michael J. Murray, Helena E. Richardson, Anthony M. Brumby

**Affiliations:** 1 Cell Cycle and Development Laboratory, Research Division, Peter MacCallum Cancer Centre, 7 St Andrew’s Place, East Melbourne, Melbourne, Victoria, Australia; 2 Bioinformatics Core Facility, Research Division, Peter MacCallum Cancer Centre, 7 St Andrew’s Place, East Melbourne, Melbourne, Victoria, Australia; 3 School of Biosciences, University of Melbourne, 1–100 Grattan Street, Parkville, Melbourne, Victoria, Australia; 4 Sir Peter MacCallum Department of Oncology, Peter MacCallum Cancer Centre, 7 St Andrew’s Place, East Melbourne, Melbourne, Victoria, Australia; 5 Department of Anatomy and Neuroscience, University of Melbourne, 1–100 Grattan Street, Parkville, Melbourne, Victoria, Australia; 6 Department of Biochemistry and Molecular Biology, University of Melbourne, 1–100 Grattan Street, Parkville, Melbourne, Victoria, Australia; 7 School of Molecular Sciences, La Trobe University, Victoria, Australia; University of Massachusetts Medical School, UNITED STATES

## Abstract

During tumorigenesis, pathways that promote the epithelial-to-mesenchymal transition (EMT) can both facilitate metastasis and endow tumor cells with cancer stem cell properties. To gain a greater understanding of how these properties are interlinked in cancers we used *Drosophila* epithelial tumor models, which are driven by orthologues of human oncogenes (activated alleles of Ras and Notch) in cooperation with the loss of the cell polarity regulator, *scribbled* (*scrib*). Within these tumors, both invasive, mesenchymal-like cell morphology and continual tumor overgrowth, are dependent upon Jun N-terminal kinase (JNK) activity. To identify JNK-dependent changes within the tumors we used a comparative microarray analysis to define a JNK gene signature common to both Ras and Notch-driven tumors. Amongst the JNK-dependent changes was a significant enrichment for BTB-Zinc Finger (ZF) domain genes, including *chronologically inappropriate morphogenesis* (*chinmo*). *chinmo* was upregulated by JNK within the tumors, and overexpression of *chinmo* with either *Ras^V12^* or *N^intra^* was sufficient to promote JNK-independent epithelial tumor formation in the eye/antennal disc, and, in cooperation with *Ras^V12^*, promote tumor formation in the adult midgut epithelium. Chinmo primes cells for oncogene-mediated transformation through blocking differentiation in the eye disc, and promoting an escargot-expressing stem or enteroblast cell state in the adult midgut. BTB-ZF genes are also required for Ras and Notch-driven overgrowth of *scrib* mutant tissue, since, although loss of *chinmo* alone did not significantly impede tumor development, when loss of *chinmo* was combined with loss of a functionally related BTB-ZF gene, *abrupt*, tumor overgrowth was significantly reduced. *abrupt* is not a JNK-induced gene, however, Abrupt is present in JNK-positive tumor cells, consistent with a JNK-associated oncogenic role. As some mammalian BTB-ZF proteins are also highly oncogenic, our work suggests that EMT-promoting signals in human cancers could similarly utilize networks of these proteins to promote cancer stem cell states.

## Introduction

The Epithelial-to-mesenchymal transition (EMT), a developmental process involved in morphogenesis, organogenesis and wound healing (reviewed in [1]), can be coopted by epithelial cancer cells to gain metastatic potential (reviewed in [2]). Over recent years, it has also become apparent that the activation of an EMT can endow cancer cells with stem cell-like properties essential for tumor maintenance (reviewed in [3]). Triggers driving the induction of EMTs during tumorigenesis are beginning to be elucidated, and can include heterotypic interactions between tumor and associated stromal cells as a result of localized inflammation [4–6]. Cytokines such as IL-6 can promote an EMT and endow tumor cells with cancer stem cell properties [7], and TGFβ, which has well-established roles in the induction of EMT, can cooperate with TNF to induce EMT, stemness and tumorigenicity [8]. Well-characterized downstream regulators of the EMT programme include transcription factors of the ZEB, Snail, Twist and NF-κB families [9–11], many of which converge upon the repression of E-cadherin expression. How this is linked to self-renewal programmes however, remains poorly understood. The acquisition of cancer stem cell properties induced by inflammation is associated with NF-κB and STAT-dependent pathways [5], however, the down regulation of E-cadherin could also help drive self-renewal through the release of β-catenin, and activation of Wnt signaling. Indeed, the loss of polarized epithelial constraints may promote self-renewal through deregulation of the Scrib cell polarity module, and subsequent activation of the Hippo negative tissue growth pathway effector, TAZ [12]. Deciphering the complex interrelationship that exists between the EMT and self-renewal pathways in cancer cells is a major challenge and will require the use of powerful model systems to tease out the separate and interconnected aspects of these two key developmental properties.

*Drosophila* is an excellent organism to model many of the complexities of human cancers [13]. We have developed “two-hit” models of epithelial cancer in the larval eye epithelium that are driven by the expression of activated alleles of either *Ras* (*Ras* ^*V12*^ or *Ras* ^*ACT*^) or *Notch* (*N* ^*intra*^ or *N* ^*ACT*^) within clones of tissue mutant for the epithelial cell polarity regulator, Scrib. These tumors show remarkable parallels to mammalian cancers, including overgrowth, failure to differentiate, invasion and metastasis [14–16]. The overgrowth and invasion in these tumors is driven by an inflammation response. Hemocytes (blood cells) are recruited to the tumor and secrete TNF, thereby activating JNK signaling within the tumor cells [17–19]. In the case of a single-hit induced by the loss of *scrib*, JNK activation induces cell death, thereby removing the aberrant cells. However, in the presence of a second hit (either *Ras* ^*ACT*^ or *N* ^*ACT*^), cell death is prevented, and instead JNK promotes tumor overgrowth and invasion [16, 20, 21]. Indeed, JNK is sufficient, when overexpressed, to cooperate with Ras^ACT^ in the formation of invasive neoplasia [22]. In the *scrib* ^-^ + *Ras* ^*V12*^ tumors, JNK-positive tumor cells with mesenchymal-like morphology are observed at the invasive front [16], and transcriptional targets of JNK in the tumors include genes required for invasion, such as *Matrix metalloproteinase 1* (*Mmp1*), *Paxillin* (*Pax*) [16, 21], and the Filamin, *cherio* (*cher*) [23]. JNK also drives tumor overgrowth by inducing the expression of IL-6 like cytokines, Unpaired1/2/3 [19, 24], which promote STAT-dependent proliferation [25, 26]. Other tumor growth promoting pathways are also likely to be activated by JNK, since JNK can promote the expression of morphogens such as Dpp (TGFβ-like) and Wingless (the *Drosophila* Wnt ligand) [27], as well as increase the activity of the Hippo pathway transducer, Yorkie (Yki) [28], which is required for tumor overgrowth [29]. JNK activation within the tumors also induces the expression of Insulin-like peptide 8 (dIlp8), which acts to prevent the production of the steroid hormone ecdysone, thereby preventing the onset of pupariation and resulting in an extending larval stage of development during which the tumor continues to overgrow [30, 31]. Thus, multiple effectors of JNK signaling collectively drive tumorigenesis in *Drosophila*.

The invasion and continual proliferation induced by JNK signaling in *Drosophila* tumors is reminiscent of cancer stem cell properties induced by EMT-promoting signals in mammalian cells. Indeed roles for JNK in mediating EMTs in *Drosophila* development have been well documented, including during dorsal closure and imaginal disc eversion, and it is likely that similar developmental pathways are induced by JNK activation in the *Drosophila* tumor cells [32]. Moreover, in other tissues, JNK activation has also been linked to promoting epithelial stem cell proliferation; tissue damage to the adult midgut promotes JNK-induced expression of Upd and related cytokines, to promote STAT-dependent intestinal stem cell proliferation and regenerative repair [33, 34]. The Ras and Notch-driven epithelial tumors develop in the eye-antennal imaginal disc which is not thought to contain epithelial stem cells, however, the eye disc does contain progenitor cells that can also overproliferate in response to excessive STAT activity [35]. Thus, similar JNK-induced developmental pathways involved in STAT-mediated stem cell proliferation and homeostasis may be operative in *Drosophila* tumors. Understanding how EMT and tumor overgrowth promoting activities are inter-linked by JNK signaling within *Drosophila* tumors therefore has the potential to provide important insight into how these properties are inter-connected in human cancer stem cells.

In this study we utilize a comparative microarray approach in *Drosophila* to identify JNK-dependent transcriptional changes in Ras and Notch-driven eye-antennal disc tumors. Our results identify the BTB-ZF family protein, Chronologically inappropriate morphogenesis (Chinmo), as a JNK-induced gene that is sufficient to block differentiation and cooperate with Ras^ACT^ and N^ACT^ in driving tumorigenesis in the eye-antennal disc. *chinmo* over-expression can also induce increased numbers of stem or enteroblast like cells in the adult midgut, and in cooperation with Ras^ACT^, induce intestinal neoplasia. The overgrowth of the Ras and Notch-driven eye-antennal disc tumors is dependent upon the partially redundant activity of Chinmo, with another functionally related BTB-ZF protein, Abrupt, which, although not identified as a JNK target, is expressed in the eye disc progenitor cells. We also identify a third BTB-ZF gene, *fruitless*, as being a JNK-induced gene within the tumors, which, similar to *chinmo*, can also cooperate with either Ras^ACT^ or N^ACT^ in promoting eye-antennal disc tumorigenesis. Thus, overall, our data indicates that the JNK-induced expression of BTB-ZF genes can be highly oncogenic, can function to maintain progenitor-like states, and are required for overgrowth in *Drosophila* epithelial tumors.

## Materials and Methods

### *Drosophila* stocks

The following *Drosophila* stocks were used: *ey-FLP1*,*UAS-mCD8-GFP;;Tub-GAL4*,*FRT82B*,*Tub-GAL80* [36]; *esg-GAL4*,*tub-GAL80* ^*ts*^ [34]; *UAS-bsk* ^*DN*^ [37]; *chinmo* ^*k13009*^ (*chinmo-lacZ*); *UAS-chinmo* ^*FL*^ [38]; *UAS-GFP*; *hth* ^*P2*^ [39]; *msn* ^06946^ (*msn-lacZ*) [40]; *UAS-N* ^*intra*^ (*UAS-N* ^*ACT*^) [41]; *UAS-phl* ^*gof*^ (*UAS-Raf* ^*gof*^) [42]; *UAS-dRas1* ^*v12*^ (*UAS-Ras* ^*ACT*^) [43]; *FRT82B*,*scrib* ^*1*^ [44]; *UAS-ab* ^*RNAi*^ (NIG #4807R-2); *UAS-ab* ^*RNAi*^ (VDRC #104582); *UAS-chinmo* ^*RNAi*^ (NIG #17156R-1 or #17156R-2); *UAS-ttk* ^*RNAi*^ (VDRC #101980); *UAS-br* ^*RNAi*^ (VDRC #104648); *UAS-fru* ^*C*^ [45]; *ptc-GAL4* [46]; *UAS-hep* ^*ACT*^ (BL#58780); *UAS-p35* (BL#5072).

### Mosaic analysis

Clonal analysis utilized MARCM (mosaic analysis with repressible cell marker) [47] with *FRT82B* and *ey*-*FLP1* to induce clones and *mCD8-GFP* expression to mark mutant tissue. All fly crosses were carried out at 25°C and grown on standard fly media unless otherwise stated.

### Analysis of adult midguts

*esg-GAL4*,*tub-GAL80* ^*ts*^ flies were crossed to the *UAS-transgene* of interest at 18°C. Progeny flies carrying *esg-GAL4*,*tub-GAL80* ^*ts*^ with the transgene of interest were collected over 5 days and stored at 18°C until shifting to 29°C, on standard food, for the specified number of days. Midguts were then harvested for immunohistochemical analysis.

### Immunohistochemistry

Imaginal discs were dissected in phosphate-buffered saline (PBS) from either wandering 3^rd^ instar larvae or from staged lays for larvae of genotypes which failed to pupate and entered an extended larval stage of development. Midguts from adult flies were also dissected in PBS. Tissues were fixed in 4% formaldehyde in PBS, and blocked in 2% goat serum in PBT (PBS 0.1% Triton X-100). Primary antibodies were incubated with the samples in block overnight at 4°C, and were used at the following concentrations; rabbit anti-Ab (S. Crews, 1/200), rabbit anti-Ato ([48], 1/1000), mouse anti-β-galactosidase (Rockland, 1/400), mouse anti-Br-core (Developmental Studies Hybridoma Bank (DSHB), 1/200), rabbit anti-Chinmo ([38], 1/500); mouse anti-Dac (DSHB, 1/10), mouse anti-Elav (DSHB, 1/20), mouse anti-Eya (DSHB, 1/20), guinea pig anti-Hth ([49], 1/100), mouse anti-Mmp1 (DSHB, 1/20), mouse anti-Pros (DSHB, 1/100), rat anti-Ttk-69 (1/200). Secondary antibodies used were; anti-mouse/rat Alexa647 (Invitrogen) and anti- rabbit Alexa488 (Invitrogen) at 1/400 dilution. F-actin was detected with phalloidin–tetramethylrhodamine isothioblueate (TRITC; Sigma, 0.3 μM, 1/1000). DNA was stained with Hoechst. Samples were mounted in 80% glycerol.

### Microscopy and image processing

All samples were analysed by confocal microscopy on an Olympus FV1000 or Leica TCS SP5 microscope. Single optical sections were selected in FluoView software before being processed in Adobe Photoshop CS2 and assembled into figures in Adobe Illustrator CS2.

### Expression array and bioinformatic analysis

Eye/antennal discs were dissected from ~5 day old larvae bearing either *FRT82B* control clones, *scrib* ^*1*^ + *Ras* ^*ACT*^ clones, *scrib* ^*1*^ + *Ras* ^*ACT*^ + *bsk* ^*DN*^ clones, *scrib* ^*1*^ + *N* ^*ACT*^ clones, or *scrib* ^*1*^ + *N* ^*ACT*^ + *bsk* ^*DN*^ clones. For the expression array, 50 pairs of discs per genotype were used to prepare RNA. Samples were prepared in triplicate, and the RNA isolated using TRIZOL, before being column purified (Qiagen). Probes were hybridized to Affymetrix GeneChip *Drosophila* Genome 2.0 Array.

The raw data was analysed using the R [50] statistical software and Bioconductor [51]. The data was loaded, background adjusted and normalized using the Affy [52] and gcRMA [53] packages and the arrays were assessed for quality using the affyPLM [54] package. Following quality assessment a linear model was fitted to the data using the LIMMA [55] package. For each contrast of interest moderated t-statistics were computed using empirical Bayes moderation of the standard errors towards a common value. The resultant p-values were adjusted for false discovery using the Benjamini & Hochberg [56] method and those with a 2 fold change and an adjusted p-value less than 0.05 were considered significant.

Expression arrays were deposited in the National Center for Biotechnology Information (NCBI) Gene Expression Omnibus (GEO) repository as CEL files, under the accession number GSE42938.

We acknowledge our use of the gene set enrichment analysis, GSEA software, and Molecular Signature Database (MSigDB) [57]. GO term enrichment was performed using AmiGO [58].

## Results

### A comparative microarray analysis identifies JNK-dependent gene expression changes in Ras and Notch-driven tumors

Previous analysis of *Ras* ^*ACT*^ and *N* ^*ACT*^-driven tumors in *Drosophila* had established that continual tumor overgrowth, migration and invasion, throughout an extended larval stage, were dependent upon JNK signaling. Blocking JNK within the tumors, through the ectopic expression of a dominant negative JNK transgene (*bsk* ^*DN*^), restored pupariation, thus curtailing tumor overgrowth during larval development, and blocked invasive cell morphology [16, 20, 21]. The diverse effects of JNK signaling within the tumor (invasion and overgrowth) are reminiscent of how EMT-promoting signals can activate cancer stem cell properties in human tumor cells. To further understand how overgrowth and mesenchymal cell behaviour could be interlinked in *Drosophila* tumor cells, we exploited a comparative microarray approach to identify, in a relatively unbiased manner, JNK-induced transcriptional changes within both Ras and Notch-driven tumors.

**Fig 1:**
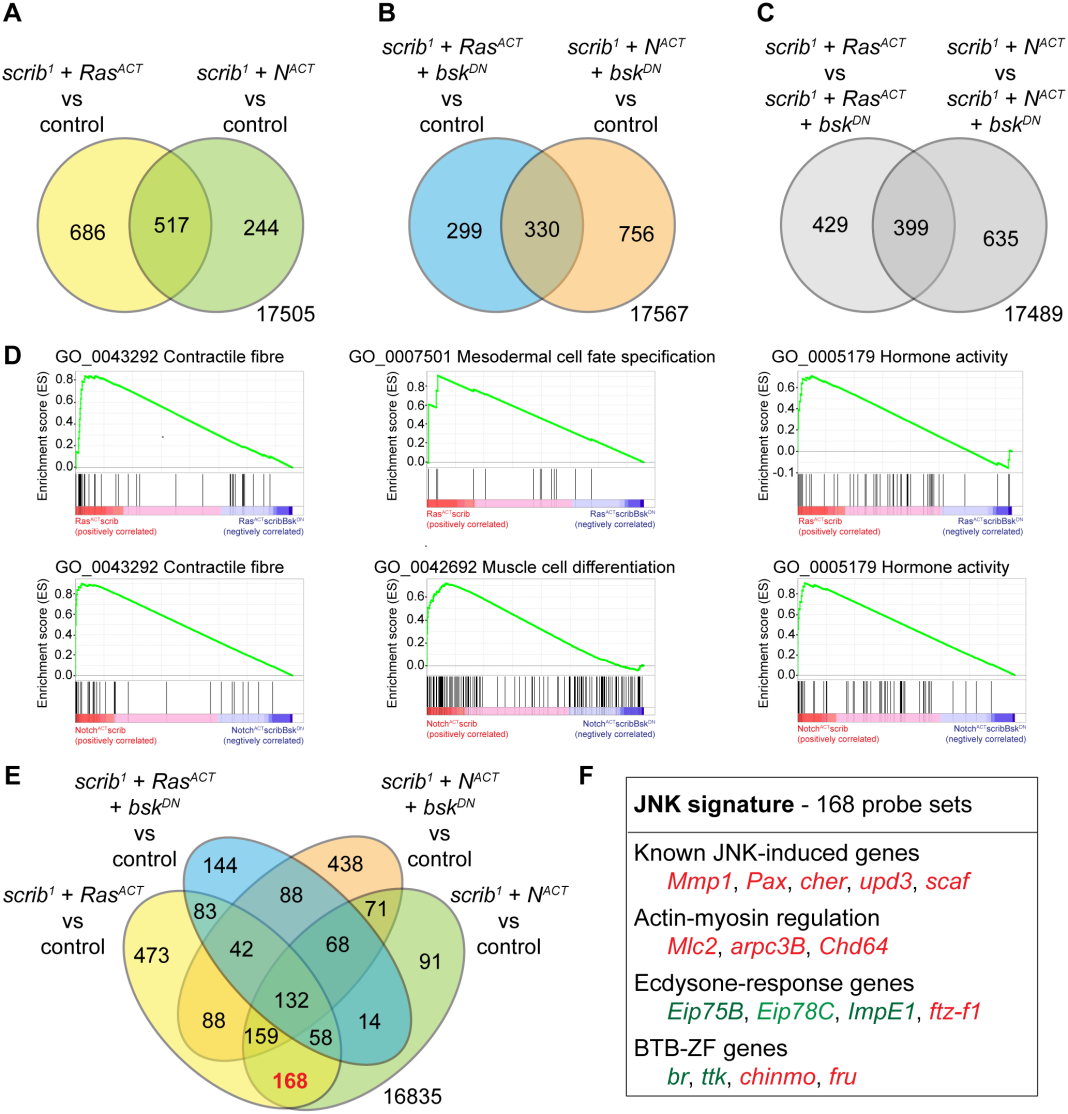
Differentially expressed genes in *scrib* ^*1*^ + *Ras* ^*ACT*^ (+/- *bsk* ^*DN*^) and *scrib* ^*1*^ + *N* ^*ACT*^ (+/- *bsk* ^*DN*^) mosaic eye-antennal discs. (A) Venn diagram showing the number of differentially expressed probes (log base 2 fold change>1 and p<0.05) in *scrib* ^*1*^ + *Ras* ^*ACT*^ and *scrib* ^-^ + *N* ^*ACT*^ mosaic eye-antennal discs compared to control *FRT82B* eye-antennal discs. (B) Venn diagram showing the number of differentially expressed probes (log base 2 fold change>1 and p<0.05) in *scrib* ^*1*^ + *Ras* ^*ACT*^ + *bsk* ^*DN*^ and *scrib* ^*1*^ + *N* ^*ACT*^ + *bsk* ^*DN*^ mosaic eye-antennal discs compared to control *FRT82B* eye-antennal discs. (C) Venn diagram showing the number of differentially expressed probes (log base 2 fold change>1 and p<0.05) in *scrib* ^*1*^ + *Ras* ^*ACT*^ and *scrib* ^*1*^ + *N* ^*ACT*^ mosaic eye-antennal discs compared to *scrib* ^*1*^ + *Ras* ^*ACT*^ + *bsk* ^*DN*^ and *scrib* ^*1*^ + *N* ^*ACT*^ + *bsk* ^*DN*^ mosaic eye-antennal discs, respectively. (D) Examples of gene sets identified by GSEA from genes differentially expressed in *scrib* ^*1*^ + *Ras* ^*ACT*^ versus *scrib* ^*1*^ + *Ras* ^*ACT*^ + *bsk* ^*DN*^ mosaic eye antennal discs (top 3 samples), and *scrib* ^*1*^ + *N* ^*ACT*^ versus *scrib* ^*1*^ + *N* ^*ACT*^ + *bsk* ^*DN*^ mosaic eye-antennal discs (bottom 3 samples). (E) Venn diagram showing the number of differentially expressed probes (log base 2 fold change>1 and p<0.05) in *scrib* ^*1*^ + *Ras* ^*ACT*^, *scrib* ^*1*^ + *N* ^*ACT*^, *scrib* ^*1*^ + *Ras* ^*ACT*^ + *bsk* ^*DN*^ and *scrib* ^*1*^ + *N* ^*ACT*^ + *bsk* ^*DN*^ mosaic eye-antennal discs compared to control *FRT82B* eye-antennal discs. (F) Selected genes identified from the 168 probes (corresponding to 103 genes) differentially expressed in *scrib* ^*1*^ + *Ras* ^*ACT*^ and *scrib* ^*1*^ + *N* ^*ACT*^ mosaic eye-antennal discs (compared to *FRT82B*), but not in *scrib* ^*1*^ + *Ras* ^*ACT*^ + *bsk* ^*DN*^ and *scrib* ^*1*^ + *N* ^*ACT*^ + *bsk* ^*DN*^ mosaic eye-antennal discs (compared to *FRT82B*). Red genes are upregulated, green genes downregulated, by JNK in the tumors.

The expression profile for mosaic eye-antennal discs of equivalent age were determined for *scrib* ^-^ + *Ras* ^*ACT*^ and *scrib* ^-^ + *N* ^*ACT*^ samples, as well as for the same genotypes expressing *bsk* ^*DN*^, and control eye-antennal discs carrying wild type clones (see Materials and Methods). Using a log base 2 fold change>1 and p<0.05 as cut-off values for significantly deregulated genes, we first compared the four tumor samples to the control discs. This revealed that 1203 probe sets were deregulated in *scrib* ^-^ + *Ras* ^*ACT*^ tumors, and 761 probe sets in *scrib* ^-^ + *N* ^*ACT*^ tumors (Fig 1A). Of these, 517 probe sets (43% of the Ras tumors, and 68% of Notch-driven tumors) were shared between the two tumor types, indicating considerable genetic similarity. Upon expressing *bsk* ^*DN*^ within the tumors, and comparing once again to control discs, 629 probe sets were deregulated in the *scrib* ^-^ + *Ras* ^*ACT*^ + *bsk* ^*DN*^ sample (with only 315, or 50%, shared with *scrib* ^-^ + *Ras* ^*ACT*^ tumors), and 1086 probe sets were deregulated in the *scrib* ^-^ + *N* ^*ACT*^ + *bsk* ^*DN*^ sample (with only 430, or 40%, shared with *scrib* ^-^ + *N* ^*ACT*^ tumors) (Fig 1B). Thus, blocking JNK exerted a major impact upon the profile of transcriptional deregulation within the tumors.

To specifically focus upon the JNK-dependent changes within the Ras and Notch-driven tumors, we next compared the expression profile of each tumor sample back to their respective *bsk* ^*DN*^-expressing samples (Fig 1C and S1 File). This showed that 828 probes were deregulated in *scrib* ^-^ + *Ras* ^*ACT*^ tumors compared to *scrib* ^-^ + *Ras* ^*ACT*^ + *bsk* ^*DN*^, and 1034 probes were significantly deregulated in *scrib* ^-^ + *N* ^*ACT*^ tumors compared to *scrib* ^-^ + *N* ^*ACT*^ + *bsk* ^*DN*^. Gene Set Enrichment Analysis (GSEA) indicated that amongst the JNK-dependent changes in both tumor types there was enrichment for functional roles consistent with the proposed roles of JNK signaling in tumor development. These included; 1) mesoderm and muscle-related gene sets (eg Contractile fibre/Myofibril/Myosin complex), consistent with cell migration and a mesenchymal-like phenotype; 2) Toll signaling/Inflammation (eg Regulation of Toll signaling pathway/Defense response/Immune response), consistent with an inflammation response and the attraction of hemocytes; and 3) Hormone signaling (eg Hormone activity), consistent with the failure of the tumor-bearing larvae to undergo an ecdysone-induced pupariation response (Fig 1D and S1 File). Thus, the JNK-dependent changes in both Ras and Notch-driven tumors exhibited significant functional overlap, and indeed, 399 probes, or close to a half of the JNK-dependent changes (48% of Ras, 39% of Notch) were shared between the two tumor types.

To validate the expression array data, we determined if previously characterized JNK-induced target genes, such as the negative regulators of the pathway, *puckered* (*puc*) [59] and *scarface* (*scaf*),[60], would be amongst the JNK-dependent changes common to both Ras and Notch-driven tumors. Indeed, both genes were upregulated by JNK within the tumors, thus confirming the arrays’ ability to identify bona fide JNK targets (S1 Table). In addition, the GSEA identification of hormone signaling reflected the repression of ecdysone response genes within the tumors, and previous studies have indicated that this is due to the JNK-induced expression of *Ilp8* (*CG14059*), which prevents the release of ecdysone [30, 31]. Consistent with this, *Ilp8* was upregulated by JNK in both tumor types (S1 Table). The GSEA also indicated significant enrichments for genes associated with motile activity and mesodermal cell fate, and indeed, four previously characterized JNK targets within *Drosophila* tumors, the *Matrix metalloproteinase 1* (*Mmp1*) [21], *Paxillin* (*Pax*) [16], *cheerio* (*cher*) [23] and *PDGF- and VEGF-receptor related* (*Pvr*) [61], were also amongst the significant JNK-dependent changes identified in the array (S1 Table), as were the PVR ligands, Pvf1 and Pvf2, which are similarly known to be induced by JNK [62]. Finally, JNK signaling within tumors induces the expression of cytokines capable of activating JAK/STAT signaling (Upd1/2/3), which is known to be required for *scrib* ^-^ + *Ras* ^*ACT*^ tumor overgrowth [25], and these genes were also induced by JNK within the tumors (S1 Table), further confirming the reliability of the expression array data.

Interestingly, however, although cytokines capable of activating JAK/STAT signaling were induced by JNK within both Ras and Notch-driven tumors, other proliferative pathways known to be associated with JNK activation were not implicated by the array data. Thus, although *Drosophila* JNK can induce the expression of growth-promoting morphogens during compensatory proliferation, including the *Drosophila* Wnt and TGFβ homologs, Wingless (Wg) and Decapentaplegic (Dpp), and JNK can also promote Yki-dependent proliferation [28], neither *dpp* and *wg*, nor Hippo pathway components (*expanded* (*ex*), *fat* (*ft*), *four-jointed* (*fj*), *Merlin (Mer*), *warts* (*wts*), *salvador* (*sav*), *yorkie* (*yki*) and *thread* (*th*)), were generally perturbed in a JNK-dependent manner (S1 Table). Furthermore, known regulators of cell cycle progression and cell growth (including the Retinoblastoma homologues, *Rbf* and *Rbf2*, *cycE*, *cycD*, *cycA*, Myc/*diminuitive* (*dm*), *E2f1*, *E2f2*) were also not significantly deregulated by JNK signaling within the tumors (S1 Table).

Also notable by their absence from the JNK-induced expression changes within the tumors were known mediators of the EMT. Although JNK targets from the array included multiple regulators of cell migration, *Drosophila* homologues of Twist (*twi*), Snail (*sna*, *esg* and *worniu*), E-cadherin (*shg*), and ZEB1/2 (*zfh1*, *zfh2*), were generally not identified as JNK-regulated genes, nor as genes that were significantly deregulated within the tumors compared to wild type control discs, although both *dorsal* (*dl*) and *Mef2*, which act with Twi and Sna to coordinate mesoderm formation in the embryo [63], were induced by JNK within the tumors (S1 Table). Other pathways known to promote EMT in *Drosophila* involve activation of the GATA factor, Serpent (*srp*), which downregulates *crumbs* (*crb*) expression [64], and inhibition of the Deleted in Colorectal Cancer (DCC) gene homologue, Frazzled (*fra*) [65, 66], a receptor for Netrins (NetA and NetB in *Drosophila*). Whilst components of these pathways did not show consistent deregulation in both tumors, *srp* levels were increased by JNK signaling in Notch-driven tumors, and *fra* was downregulated by JNK in Ras-driven tumors (S1 Table). It is therefore possible that multiple developmental programs, including yet to be identified ones, may cooperate to promote mesenchymal behaviour in response to JNK signaling.

### The effects of JNK signaling upon eye-antennal disc differentiation in Ras and Notch-driven tumors

We next examined whether JNK signaling influenced the differentiation state of the tumors, since it was possible that the JNK-induced proliferation and mesenchymal behaviour of the tumor cells was also associated with a progenitor-like, or cancer stem cell-like, state. Indeed, although *Drosophila* eye antennal discs are not known to contain stem cells, they do contain progenitor cells that can overproliferate in response to STAT activity [35]. Previously, we showed that although *scrib* ^-^ + *Ras* ^*ACT*^ eye disc tumors failed to express the photoreceptor differentiation marker, Elav (Embryonic lethal abnormal vision), and that blocking JNK (by expressing *bsk* ^*DN*^) was associated with a restoration to Elav expression [16], surprisingly, blocking JNK in *scrib* ^-^ + *N* ^*ACT*^ tumors failed to restore Elav expression [16]. This indicated that curtailing tumor overgrowth was not necessarily associated with restoring Elav. The expression array further validated these observations by showing Elav expression was downregulated in both tumor samples, but only restored by blocking JNK signaling in the Ras-driven tumors (Table 1). However, Elav is expressed relatively late with respect to cell fate commitment, and only in cells committed to a photoreceptor fate. It was therefore possible that the continual overgrowth of Ras and Notch-driven tumors was characterized by the failure to upregulate earlier-acting cell fate commitment regulators, and/or the continued expression of progenitor cell markers, and that the expression of these would be normalized by blocking JNK signaling. To determine if this was the case, we used the array data to examine the expression of other markers of cell fate commitment and progenitor cell states in the eye-antennal disc. Differentiation factors included the proneural factor Atonal (Ato), which is expressed just before Elav, and Sine oculis (So), Dachshund (Dac), Eyes absent (Eya), Distal antenna (Dan) and Distal antenna-related (Danr), the expression of which all precede Ato (reviewed in [67]). Dan and Danr are also expressed during antennal disc differentiation, together with the homeodomain protein Distal-less (Dll). Markers of progenitor cells in the eye disc included Homomthorax (Hth), which is downregulated as cells upregulate Dac and Eya, Teashirt (Tsh), Eyeless (Ey), Twin of eyeless (Toy) and Optix.

**Table 1:** Expression levels of eye-antennal cell fate markers in *scrib* ^*1*^ + *Ras* ^*ACT*^ and *scrib* ^*1*^ + *N* ^*ACT*^ tumors (+/- *bsk* ^*DN*^) compared to control *FRT82B* eye-antennal discs.

		Log fold change in expression compared to *FRT82B* control			
Gene	Probe set ID	*scrib* ^-^ + *Ras* ^*ACT*^	*scrib* ^-^ + *Ras* ^*ACT*^ + *bsk* ^*DN*^	*scrib* ^-^ + *N* ^*ACT*^	*scrib* ^-^ + *N* ^*ACT*^ + *bsk* ^*DN*^
*ato*	1640868_at	-1.638862	-1.405547	-1.873315	-2.57942
*dac*	1633341_s_at	-0.882682	-1.311069	-0.789177	-2.080069
*dan*	1639221_at	-0.877041	-0.830498	-1.232786	-2.413103
*danr*	1624225_at	-0.900586	-0.388284	-1.236118	-2.253406
*Dll*	1636088_at	-1.425958	-0.694563	-0.959602	-1.769509
	1630237_a_at	-1.678097	-0.698569	-1.21385	-1.844916
	1625771_at	-2.396353	n.s.	n.s.	n.s.
*elav*	1636615_at	-0.611124	0.706356	-0.838166	-1.54344
*eya*	1623206_a_at	-0.954342	-0.219169	-1.045207	-1.937237
*so*	1641675_at	-0.914148	n.s.	-0.592477	-0.97469
*ey*	1628973_a_at	-0.622696	-1.43668	n.s.	0.025858
*hth*	1632177_at	-1.617727	-0.900981	n.s.	0.752602
	1631524_a_at	-1.48697	-0.647331	-0.435273	0.41521
*optix*	1640296_a_at	-0.826046	-2.089094	n.s.	-1.675348
*toy*	1633094_a_at	-1.354635	-1.359693	n.s.	0.923041
	1623314_at	-0.656699	-1.310195	n.s.	1.538481
	1633512_at	-1.341363	-1.564924	n.s.	0.61131
*tsh*	1626150_at	-1.0428	-1.130558	n.s.	n.s.

n.s. = not significant (p value>0.05)

Interestingly, all six markers of eye-antennal cell fate commitment (*ato*, *dac*, *dan*, *danr*, *Dll*, *eya* and *so*) were downregulated within both Ras and Notch-driven tumors (Table 1). This indicated that tumor overgrowth was indeed associated with a failure to differentiate. However, blocking JNK within *scrib* ^-^ + *Ras* ^*ACT*^ and *scrib* ^-^ + *N* ^*ACT*^ tumors, by co-expressing *bsk* ^*DN*^, failed to increase *ato*, *dac*, *dan* and *so* expression in either Ras or Notch-dependent tumors; and although *danr*, *eya* and *Dll* levels were marginally increased in Ras-driven tumors expressing *bsk* ^*DN*^, their levels remained downregulated in *scrib* ^-^ + *N* ^*ACT*^ + *bsk* ^*DN*^ discs (Table 1). This suggested that blocking JNK within Ras and Notch-driven tumors was not acting through a common pathway to restore differentiation to the tumor cells. Furthermore, although we hypothesized that progenitor state markers might be increased within the tumors if JNK signaling was promoting their expression to maintain tumor overgrowth, the expression array data indicated that *hth*, *tsh*, *ey*, *toy* and *optix* expression were either not significantly altered, or were downregulated, in both Ras and Notch-driven tumors compared to control eye-antennal discs. In fact, blocking JNK signaling in Notch tumors was associated with an upregulation of both *hth* and *toy* expression compared to control mosaic discs. Therefore, it did not appear likely that JNK was promoting Ras and Notch-driven tumor overgrowth by maintaining a progenitor cell state characterized by the expression of *hth* and other known progenitor cell-expressing factors.

To confirm these data, and to specifically observe whether the tumor cells failed to express these proteins, we examined mosaic eye-antennal discs using immunohistochemical analysis with available antibodies directed against the products of three of the differentiation genes, Eya, Dac and Ato, and the progenitor state marker, Hth. This analysis again validated the expression array data, since all three differentiation markers were repressed in *scrib* ^-^ + *Ras* ^*ACT*^ (S1 Fig) and *scrib* ^-^ + *N* ^*ACT*^ (S2 Fig) tumor tissue, and remained repressed in *scrib* ^-^ + *Ras* ^*ACT*^ + *bsk* ^*DN*^ and *scrib* ^-^ + *N* ^*ACT*^ + *bsk* ^*DN*^ clones, except Eya, the levels of which were increased within in *scrib* ^-^ + *Ras* ^*ACT*^ + *bsk* ^*DN*^ clones (S1H Fig). Similarly, in Ras-driven tumors, the progenitor state factor Hth was also repressed (S3B Fig), consistent with the array data. In contrast, we observed that Hth levels in Notch-driven tumors were increased in posteriorly localized cells that would normally have downregulated Hth, consistent with the maintenance of a progenitor cell state (S3C Fig). However, *scrib* ^-^ + *N* ^*ACT*^ + *bsk* ^*DN*^ clones also exhibited elevated Hth levels indicating that the upregulation was not JNK-dependent (S3D Fig); and expressing *N* ^*ACT*^ in *scrib* ^-^ *hth* ^-^ double mutant clones still elicited the formation of large invasive tumors throughout an extended larval stage, indicating that Hth was not absolutely required for tumor overgrowth (S3F Fig). Thus, we conclude that, although both Ras and Notch-driven tumors fail to transition to Dac/Eya expression, the JNK-dependent maintenance of an Hth-dependent progenitor state, was not likely to be key to their continual overgrowth.

### The expression of BTB-ZF genes are deregulated by JNK in Ras and Notch-driven tumors

The analysis of cell fate markers in the *Drosophila* eye-antennal disc indicated that tumor overgrowth was associated with a block to differentiation, but failed to identify specific JNK-effectors, common to both Ras and Notch-driven tumors, that could be involved with maintaining a progenitor-like cell fate. To determine, in a less biased manner, if any other progenitor state factors could be induced by JNK signaling within the tumors, we further mined the array data by narrowing down the list of candidate genes. We did this by not only assuming that key JNK effectors would be common to both Ras and Notch-driven tumors, but also, that upon blocking JNK activity, the expression of these candidates would be normalized to approximately wild type levels. In other words, that by comparing the expression profiles of all four tumorigenic and non-tumorigenic samples back to wild type control discs, these genes would only be significantly deregulated (log base 2 fold change>1, p<0.05) in Ras and Notch-driven tumors, but not in the non-tumorigenic samples expressing *bsk* ^*DN*^ (Fig 1E). This four-way comparison yielded groupings of probes specifically deregulated by *Ras* ^*ACT*^, but not *N* ^*ACT*^, expression (eg. *EGFR*, *sty*), or by *N* ^*ACT*^, but not *Ras* ^*ACT*^, expression (eg. *HLHm3*, *Ser*, *neur*), as well as generating a focussed list of 168 probe sets, corresponding to 103 genes, deregulated by JNK and common to both Ras and Notch-dependent tumors (32 genes downregulated; 71 genes upregulated by JNK; Fig 1E and S2 File). Significantly, this JNK signature included *Mmp1*, *Pax*, *cher* and *Upd3*, as well as other genes known to be induced by JNK activation in other developmental contexts (eg *scaf* [60]), and genes potentially involved in the invasive phenotype (eg the ARP family member, *Actin-related protein 2/3 complex subunit 3B* (*Arpc3B*), and *myosin light chain 2* (*Mlc2*) [68]).

Of note, from this list of 103 genes, there was a high enrichment for genes belonging to the BTB-ZF family (4 identified, out of a genome encoding 15 family members): *broad* (*br*) and *tramtrack* (*ttk*) were repressed in response to JNK signaling, whereas *fruitless* (*fru*) and *chronologically inappropriate morphogenesis* (*chinmo*) were upregulated in a JNK-dependent manner (S2 Table). BTB-ZF proteins are increasingly implicated in the aetiology of human cancers, as both tumor suppressors and oncogenes, and are also highly oncogenic in *Drosophila* when ectopically over-expressed. Our own previous screening for *Drosophila* oncogenes identified the BTB-ZF gene *abrupt*, as a potent inducer of tumorigenesis when ectopically overexpressed in *scrib* mutant cells [69]. Although *ab* was not significantly deregulated by JNK in the expression arrays (S2 Table), the BTB-ZF protein Chinmo bears many striking similarities to the functional activity of Ab. This includes both being targets of *let-7* mediated repression, and also regulating the temporal differentiation of neural cells in the brain [38, 70–72]. Furthermore, previous analysis had indicated that *chinmo* expression is associated with a stem cell state; its ectopic overexpression can promote stem cell proliferation, and, in response to JAK/STAT signaling, *chinmo* is expressed within the progenitor domain of the eye disc and required for eye disc growth and/or proliferation [26, 73]. Together, these data suggested that Chinmo could be an important STAT effector of progenitor cell maintenance in the eye-antennal disc tumors, downstream of JNK signaling.

**Fig 2:**
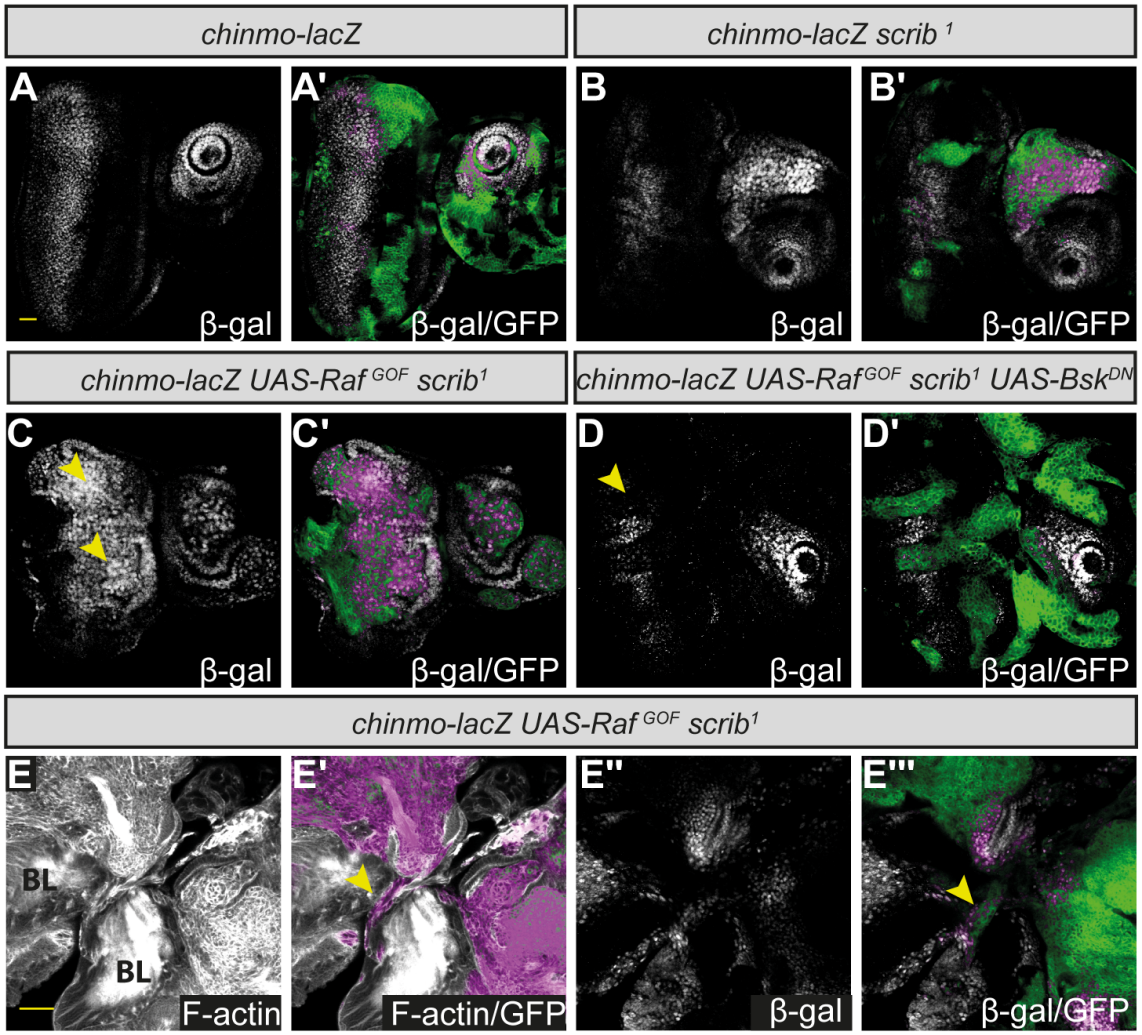
The ectopic expression of *chinmo*-*lacZ* in *scrib* ^*1*^ + *Raf* ^*gof*^ tumor cells is dependent upon JNK signaling. Mosaic eye-antennal discs, anterior to the right. Clones are generated with *ey-FLP*, and are positively marked by GFP (green, or magenta when overlaid with white). *chinmo-lacZ* expression is shown by antibody detection of β-Galactosidase (white, or magenta when overlaid with GFP in the merges). Brain lobes (BL) remain attached to the eye discs in E, and in E and E’ tissue morphology is shown with Phalloidin staining to highlight F-actin (white). Yellow scale bar corresponds to 40μm. (A) In control *FRT82B* eye-antennal discs, *chinmo-lacZ* is expressed in the centre of the antennal disc and in the posterior half of the eye disc. (B) In *scrib* ^*1*^ mosaic discs, *chinmo-lacZ* is ectopically expressed in some mutant antennal disc clones (arrowhead). (C) In *scrib* ^*1*^ + *Raf* ^*gof*^ mosaic discs, *chinmo-lacZ* is ectopically expressed in mutant clones in the antennal and eye disc (arrowheads). (D) Expressing *UAS-bsk* ^*DN*^ in *scrib* ^*1*^ + *Raf* ^*gof*^ clones abrogates the ectopic expression of *chinmo-lacZ* in the mutant clones of tissue (arrowhead). (E) In *scrib* ^*1*^ + *Raf* ^*gof*^ tumors, *chinmo-lacZ* is ectopically expressed in the tumor cells that appear to be migrating between the brain lobes (arrowhead, E’ and E’’’).

To confirm that the expression of *chinmo* was upregulated by JNK within the tumors, we examined the activity of a previously characterized enhancer trap reporter for *chinmo* expression, *chinmo-lacZ* [73]. As the reporter was inserted on the same chromosome as the *Ras* ^*ACT*^ and *N* ^*ACT*^ transgenes, we facilitated this analysis by examining *Raf* ^*gof*^-driven tumors (+/- *bsk* ^*DN*^), since the *Raf* ^*gof*^ transgene was on a different chromosome to the *chinmo-lacZ* reporter, and we had previously shown that *Raf* ^*gof*^ mimics the effects of *Ras* ^*ACT*^ in driving *scrib* ^-^ tumor overgrowth [14]. In wild type discs, *chinmo-lacZ* was expressed in the centre of the antennal disc, and also in the posterior cells of the eye disc (Fig 2A). However, in *scrib* ^-^ + *Raf* ^*gof*^ tumors, *chinmo-lacZ* was ectopically expressed within the tumor cells (Fig 2C). Furthermore, the reporter was also expressed in basally located cells that had dropped out of the epithelium, and in cells that appeared to be migrating between the brain lobes (Fig 2E), previous analysis of which had indicated are JNK positive [16]. However, upon expressing *bsk* ^*DN*^ within the *scrib* ^-^ + *Raf* ^*gof*^ tumors, the expression of *chinmo-lacZ* was normalized, consistent with it’s expression being JNK-dependent (Fig 2D).

### Overexpression of *chinmo* is sufficient to cooperate with *Ras* ^*ACT*^ or *N* ^*ACT*^ and drive JNK-independent tumor overgrowth in the eye-antennal disc

**Fig 3:**
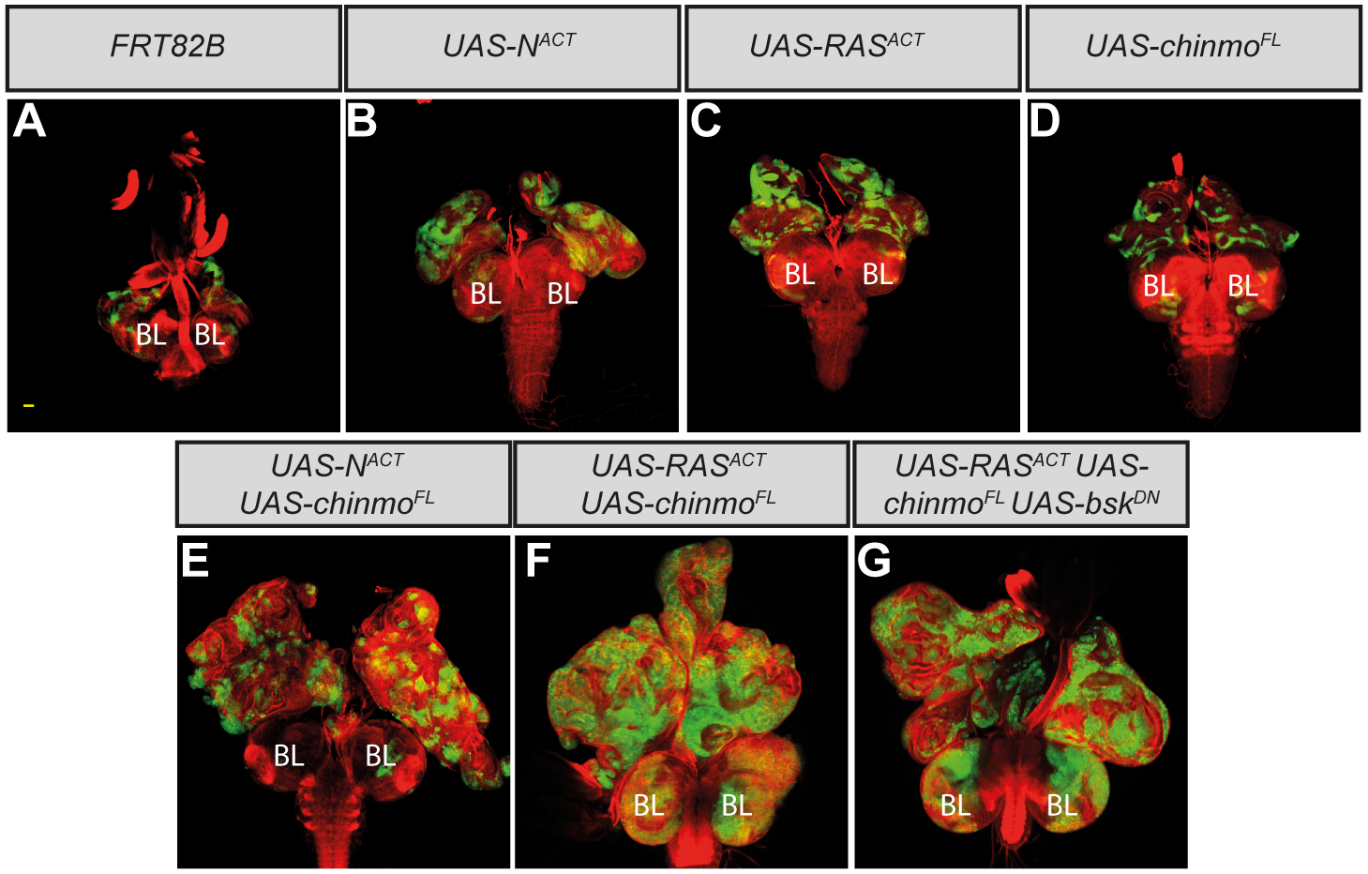
*chinmo* overexpression in eye-antennal disc clones cooperates with *Ras* ^*ACT*^ or *N* ^*ACT*^ to produce massive tumors. Larval mosaic eye-antennal discs attached to brain lobes (BL), anterior to the top, at day 5 (A-D) and day 9 (E-G). Clones are generated with *ey-FLP*, and are positively marked by GFP (green). Tissue morphology is shown with Phalloidin staining to highlight F-actin (red). The yellow scale bar corresponds to 40μm. (A-D) Control *FRT82B* eye-antennal disc clones (A), *UAS-N* ^*ACT*^-expressing clones (B), *UAS-Ras* ^*ACT*^-expressing clones (C) and *UAS-chinmo* ^*FL*^-expressing clones (D) are relatively normal in size at day 5 prior to pupariation. (E-F) Co-expressing *UAS-chinmo* ^*FL*^ with *UAS-N* ^*ACT*^ (E) or *UAS-Ras* ^*ACT*^ (F) in eye-antennal disc clones blocks pupariation, and the clonal tissue massively overgrows throughout an extended larval stage of development. Clones of mutant tissue within the brain lobes also over-proliferate to greatly enlarge the brain lobes (F). (G) Co-expressing *UAS-bsk* ^*DN*^ in *chinmo* ^*FL*^ + *Ras* ^*ACT*^ clones does not restore pupariation to the tumor-bearing larvae, and the mutant tissue overgrows throughout an extended larval stage of development.

If Chinmo acts as an important oncogenic mediator of JNK signaling, its ectopic expression might be sufficient to drive tumor overgrowth in cooperation with Ras^ACT^ or N^ACT^. To determine if this was the case, we used a transgene to ectopically express a full-length version of *chinmo*, both alone and in combination with *Ras* ^*ACT*^ or *N* ^*ACT*^ in eye disc clones. Strikingly, whereas the overexpression of *chinmo* alone did not prevent organismal pupariation and clones did not overgrow, larvae overexpressing *chinmo* with either *Ras* ^*ACT*^ or *N* ^*ACT*^ often failed to enter pupariation, and massive tumor overgrowth ensued throughout an extended larval stage (Fig 3). The brain lobes were also markedly enlarged in Ras-driven tumors, consisting of masses of clonal tissue, suggestive of excessive neuroepithelial proliferation (S4 Fig). Thus *chinmo* is sufficient to drive tumorigenesis in cooperation with ectopic Ras or Notch signaling.

The capacity of *chinmo* overexpression to cooperate with Ras or Notch in tumorigenesis is consistent with *chinmo* being an important tumorigenic effector downstream of JNK. We therefore hypothesized that the overgrowth of *chinmo*-driven tumors throughout an extended larval stage could be independent of JNK activity. Indeed, the overgrown eye-antennal discs of *chinmo* + *Ras* ^*ACT*^ or *chinmo* + *N* ^*ACT*^ tumors appeared benign, since they remained as separate entities and did not fuse to the brain lobes, consistent with a failure to activate a JNK-dependent invasion pathway. Furthermore, blocking JNK signaling within *chinmo* + *Ras* ^*ACT*^ tumors by coexpressing *bsk* ^*DN*^ in the mutant clones failed to restore pupariation to the tumor-bearing larvae, and the tumors continued to grow throughout an extended larval stage (Fig 3G). Thus, unlike *scrib* ^-^ + *Ras* ^*ACT*^/*N* ^*ACT*^ tumors, JNK signaling is not essential for *chinmo*-driven tumorigenesis.

### *chinmo* overexpression is sufficient to block epithelial differentiation in the larval eye disc and promote stem cell/enteroblast overproliferation and cooperation with *Ras* ^*ACT*^ in the adult midgut

**Fig 4:**
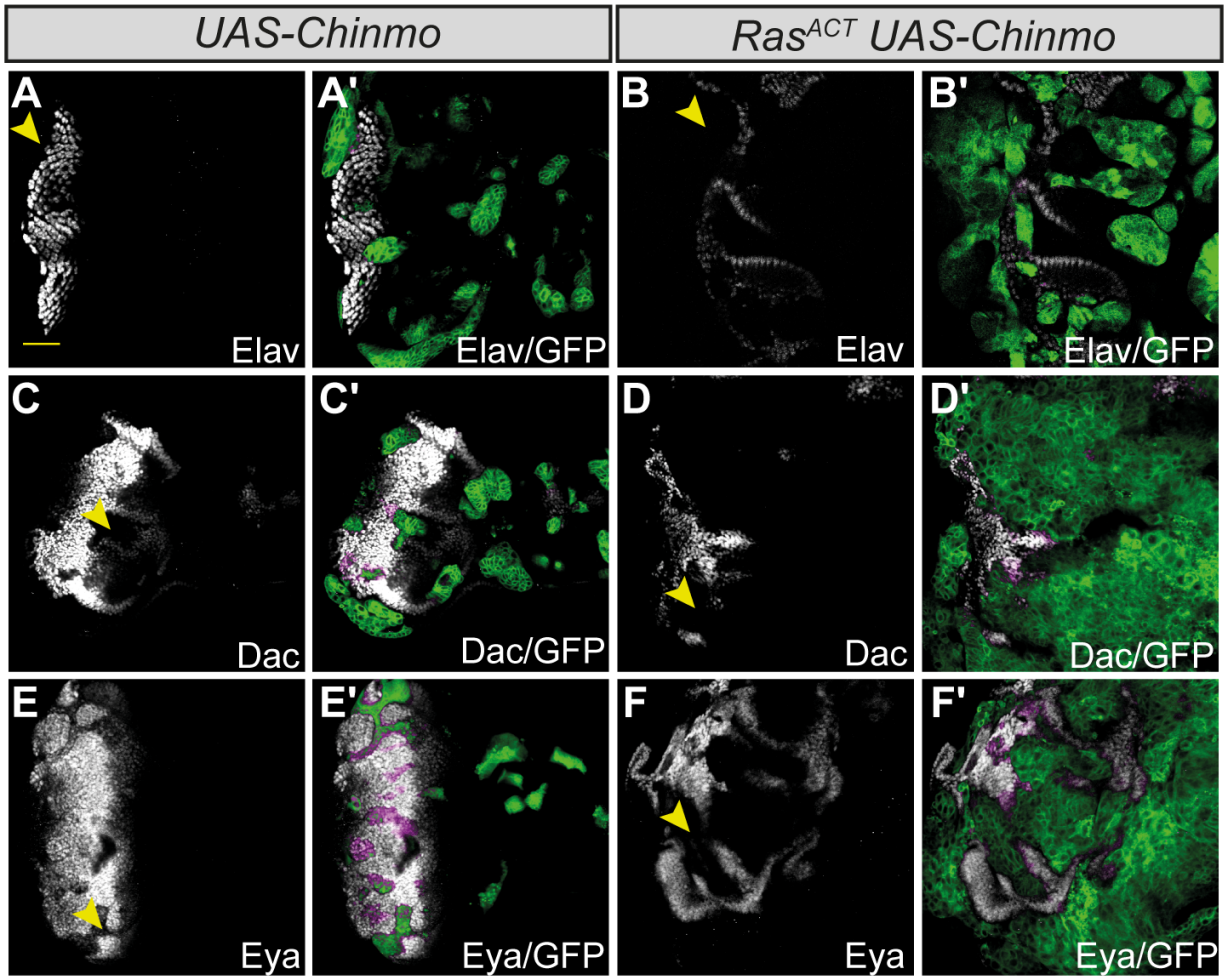
*chinmo* ^*FL*^ over-expression in clones blocks differentiation in the eye-antennal disc. Mosaic eye-antennal discs, anterior to the right. Clones are generated with *ey-FLP*, and are positively marked by GFP (green, or magenta when overlaid with white). Cell fate is marked by the expression of Elav, Dac and Eya (white, or magenta when overlaid with GFP in the merges). Yellow scale bar corresponds to 40μm. (A-F) Expressing *UAS-chinmo* ^*FL*^ in eye-antennal disc clones blocks expression of Elav (A), Dac (C) and Eya (E), and this block is maintained in *chinmo* ^*FL*^ + *Ras* ^*ACT*^ tumors (B, D, F; arrowheads).

Previous studies have revealed that *chinmo* expression is associated with progenitor-like states in some *Drosophila* tissues: it is involved in stem cell maintenance in the cyst cells of the *Drosophila* testis, as well as functioning within a heterochronic pathway controlling the timing of neural differentiation in the brain [38, 70, 73]. To determine if its oncogenic ability in the eye-antennal disc was also likely to be associated with roles in maintaining a progenitor-like state, we examined the expression of cell fate markers in *chinmo*-expressing clones. This revealed that the over-expression of *chinmo* alone was sufficient to block the expression of Dac, Eya and Elav in the eye disc (Fig 4). Similarly, *chinmo* + *Ras* ^*ACT*^ tumors were also characterized by the failure to express the differentiation factors, Dac, Eya and Elav (Fig 4). The data are therefore consistent with Chinmo functioning to prime cells towards transformation by blocking differentiation.

**Fig 5:**
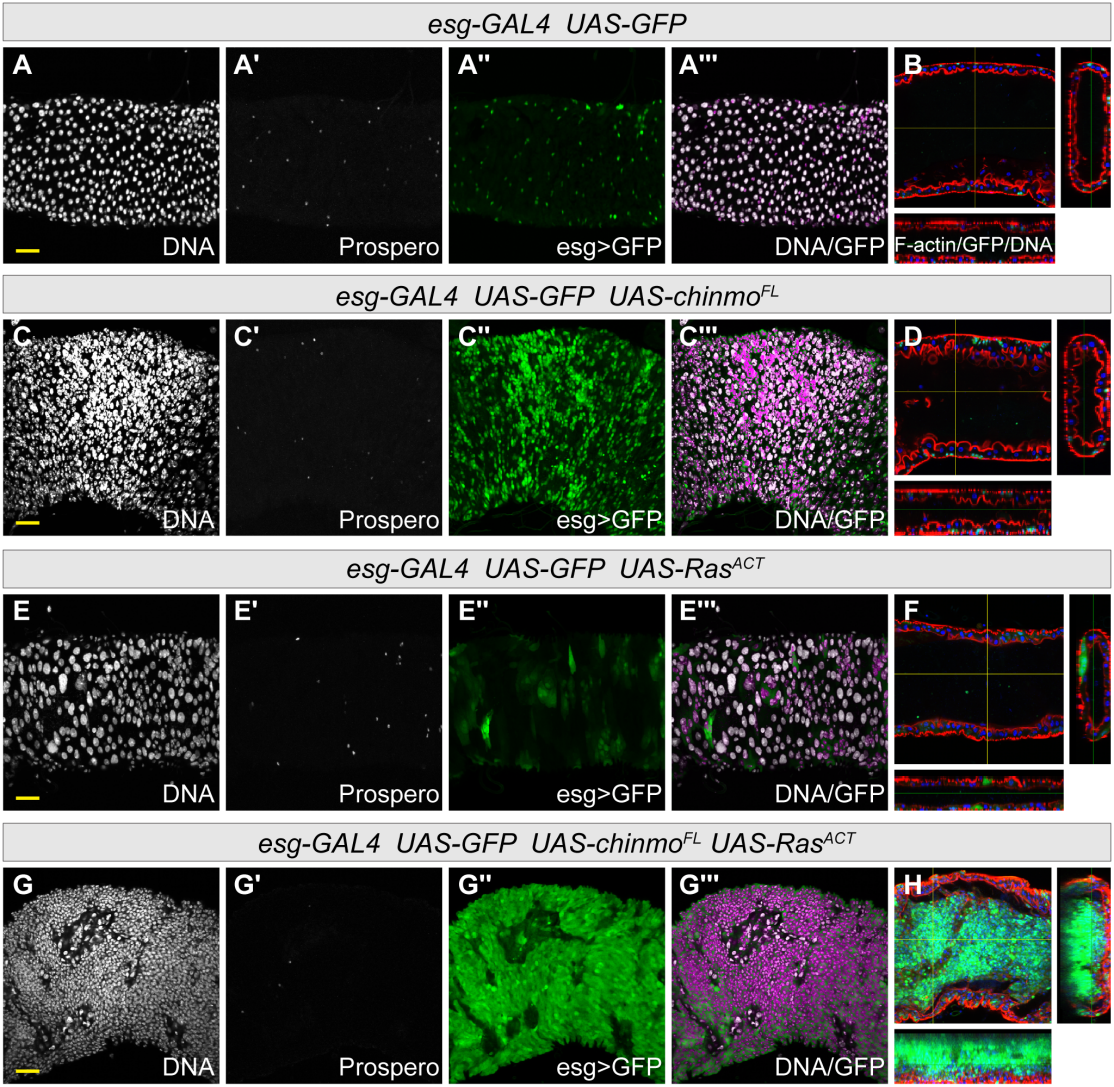
*chinmo* overexpression increases *esg>GFP* cells in the midgut, and cooperates with *Ras* ^*ACT*^ to promote midgut tumorigenesis. Sections of adult midguts expressing transgenes at 29°C under the control of *esg-GAL4*,*tub-GAL80* ^*ts*^. DNA is stained with Hoechst (white), enteroendocrine cells are identified by Prospero expression (white), and *UAS-GFP* (green) is expressed in the stem cells and enteroblasts. The overlay of DNA with GFP appears magenta in the merge. Cross sections through the centre of the intestine, stained with Phalloidin to detect F-actin (red) and Hoechst (blue) are shown in B, D, F and H. Yellow scale bar corresponds to 20μM. (A,B) Control midguts expressing *UAS-GFP* for 5 days at 29°C. (C,D) The expression of *UAS-chinmo* ^*FL*^ for 10 days at 29°C greatly increases the number of *esg>GFP* cells, whilst the number of enteroendocrine cells appears unchanged. See S5 Fig for quantifications at day 5 and 10. (E,F) Expression of *UAS-Ras* ^*ACT*^ for 7 days at 29°C induces changes in the appearance of *the esg>GFP* cells, but does not lead to the formation of tumors. (G,H) Coexpression of *UAS-chinmo* ^*FL*^ with *UAS-Ras* ^*ACT*^ for 7 days at 29°C leads to *esg>GFP* cells overtaking the entire midgut, filling the lumen of the intestine.

Since the eye-antennal disc is not a good model for investigating stem cell properties, we turned to an epithelial tissue that is maintained by stem cell divisions, the midgut of the adult fly. Using *esg-GAL4*, which is expressed in the epithelial stem cells and their progeny, the enteroblast (prior to the enteroblast differentiating into either an enterocyte and enteroendocrine cell) [74, 75], we overexpressed *chinmo* ^*FL*^ for 5–7 days in adult flies. Strikingly, this significantly increased the number of GFP positive stem cells/enteroblasts within the epithelium, suggesting that ectopic expression of *chinmo* was able to promote their proliferation. To determine if this also predisposed cells to transformation, we next coexpressed *chinmo* ^*FL*^ with *Ras* ^*ACT*^. *Ras* ^*ACT*^ does not produce tumors in the adult midgut [76], however, combined expression of *chinmo* ^*FL*^ + *Ras* ^*ACT*^ produced massive overgrowth of *esg*>*GFP* expressing cells that filled the lumen of the midgut (Fig 5 and S5 Fig), eventually causing host lethality. Thus, the ectopic expression of *chinmo* can maintain an epithelial stem cell or enteroblast state, which primes cells for transformation by Ras^ACT^.

### Chinmo, and the functionally-related BTB-ZF protein, Abrupt are required for *scrib* ^-^ + *Ras* ^*ACT*^/*N* ^*ACT*^ tumor overgrowth

**Fig 6:**
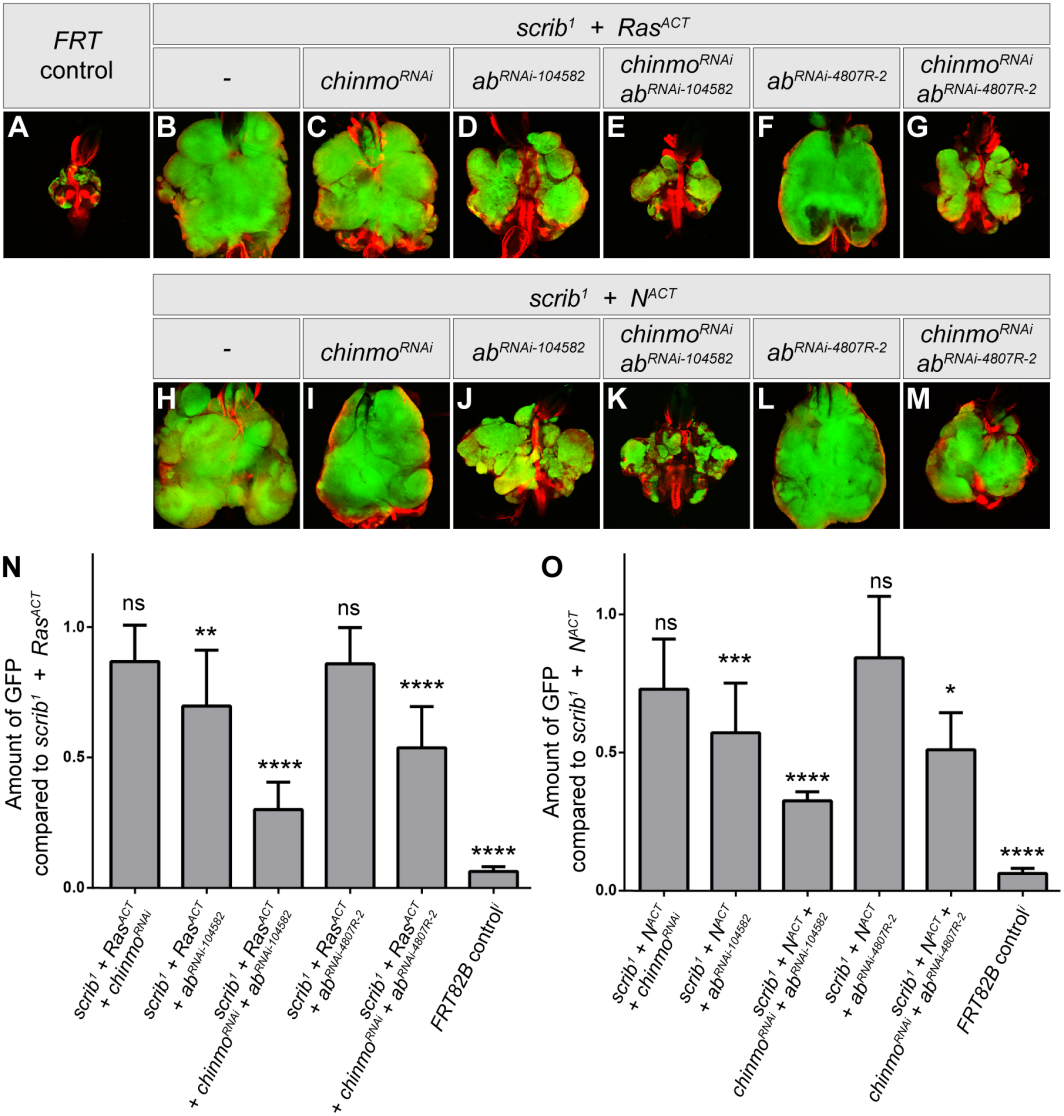
Chinmo and Abrupt are required for Ras^ACT^ and N^ACT^-driven tumor overgrowth. Mosaic eye-antennal discs, attached to brain lobes, anterior to the top, at day 5 (A) and day 9 (B-M), from larvae bearing clones of *FRT82B* (A), *scrib* ^1^ + *Ras* ^*ACT*^ (B), *scrib* ^1^ + *Ras* ^*ACT*^ + *UAS-chinmo* ^*RNAi*^ (#17156R-1) (C), *scrib* ^1^ + *Ras* ^*ACT*^ + *UAS-ab* ^*RNAi*^ (#104582) (D), *scrib* ^1^ + *Ras* ^*ACT*^ + *UAS-chinmo* ^*RNAi*^ (#17156R-1) + *UAS-ab* ^*RNAi*^ (#104582) (E), *scrib* ^1^ + *Ras* ^*ACT*^ + *UAS-ab* ^*RNAi*^ (#4807R-2) (F), *scrib* ^1^ + *Ras* ^*ACT*^ + *UAS-chinmo* ^*RNAi*^ (#17156R-1) + *UAS-ab* ^*RNAi*^ (#4807R-2) (G), *scrib* ^1^ + *N* ^*ACT*^ (H), *scrib* ^1^ + *N* ^*ACT*^ + *UAS-chinmo* ^*RNAi*^ (#17156R-1) (I), *scrib* ^1^ + *N* ^*ACT*^ + *UAS-ab* ^*RNAi*^ (#104582) (J), *scrib* ^1^ + *N* ^*ACT*^ + *UAS-chinmo* ^*RNAi*^ (#17156R-1) + *UAS-ab* ^*RNAi*^ (#104582) (K), *scrib* ^1^ + *N* ^*ACT*^ + *UAS-ab* ^*RNAi*^ (#4807R-2) (L), *scrib* ^1^ + *N* ^*ACT*^ + *UAS-chinmo* ^*RNAi*^ (#17156R-1) + *UAS-ab* ^*RNAi*^ (#4807R-2) (M). Clones are generated with *ey-FLP*, and are positively marked by GFP (green). Tissue morphology is shown with Phalloidin staining to highlight F-actin (red). (N,O) Quantification of GFP from images as represented in (A-M), normalized to either *scrib* ^1^ + *Ras* ^*ACT*^ (N) or *scrib* ^1^ + *N* ^*ACT*^ (O). Note that the quantification of GFP is based upon the amount of GFP in two-dimensional sections, as opposed to volumetric calculations of the entire tumor mass in three dimensions. It thus underestimates the true extent of tumor size reduction. n for each genotype: *FRT82B* = 6; *scrib* ^-^ + *Ras* ^*ACT*^ = *7*, + *chinmo* ^*RNAi-17156R-1*^ = 6, + *ab* ^*RNAi-104582*^ = 9, + *chinmo* ^*RNAi-17156R-1*^ + *ab* ^*RNAi-104582*^ = 6, + *ab* ^*RNAi-4807R-2*^ = 7, + *chinmo* ^*RNAi-17156R-1*^ + *ab* ^*RNAi-4807R-2*^ = 12; *scrib* ^-^ + *N* ^*ACT*^ = 10, + *chinmo* ^*RNAi-17156R-1*^ = 6, + *ab* ^*RNAi-104582*^ = 9, + *chinmo* ^*RNAi-17156R-1*^ + *ab* ^*RNAi-104582*^ = 4, + *ab* ^*RNAi-4807R-2*^ = 5, + *chinmo* ^*RNAi-17156R-1*^ + *ab* ^*RNAi-4807R-2*^ = 2. Error bars are 95% Confidence Intervals (CI). **** p < 0.0001; *** p = 0.0001 to 0.001; ** p = 0.001 to 0.01; * p = 0.01 to 0.05; ns = not significant.

Our analysis of *chinmo* overexpression had confirmed its sufficiency to promote tumorigensis in cooperation with *Ras* ^*ACT*^ or *N* ^*ACT*^ in different epithelial tissues, and the data were consistent with the possibility of *chinmo* functioning downstream of JNK in *scrib* ^-^ + *Ras* ^*ACT*^/*N* ^*ACT*^ tumors to promote overgrowth. To determine if *chinmo* does play a role in the development of *scrib* ^-^ + *Ras* ^*ACT*^/*N* ^*ACT*^ tumors, we used an RNAi transgene to knockdown *chinmo* levels. Immuno-histochemical analysis of Chinmo protein in the wing confirmed that the overexpression of *chinmo* ^RNAi^ significantly reduced Chinmo levels (S6 Fig). However, the ectopic expression of *chinmo* ^RNAi^ in either *scrib* ^-^ + *Ras* ^*ACT*^ or *scrib* ^-^ + *N* ^*ACT*^ tumors exerted little effect upon the size or invasive properties of tumors at day 9 (Fig 6). This suggested that *chinmo* was not significantly rate-limiting for invasive, tumor overgrowth.

**Fig 7:**
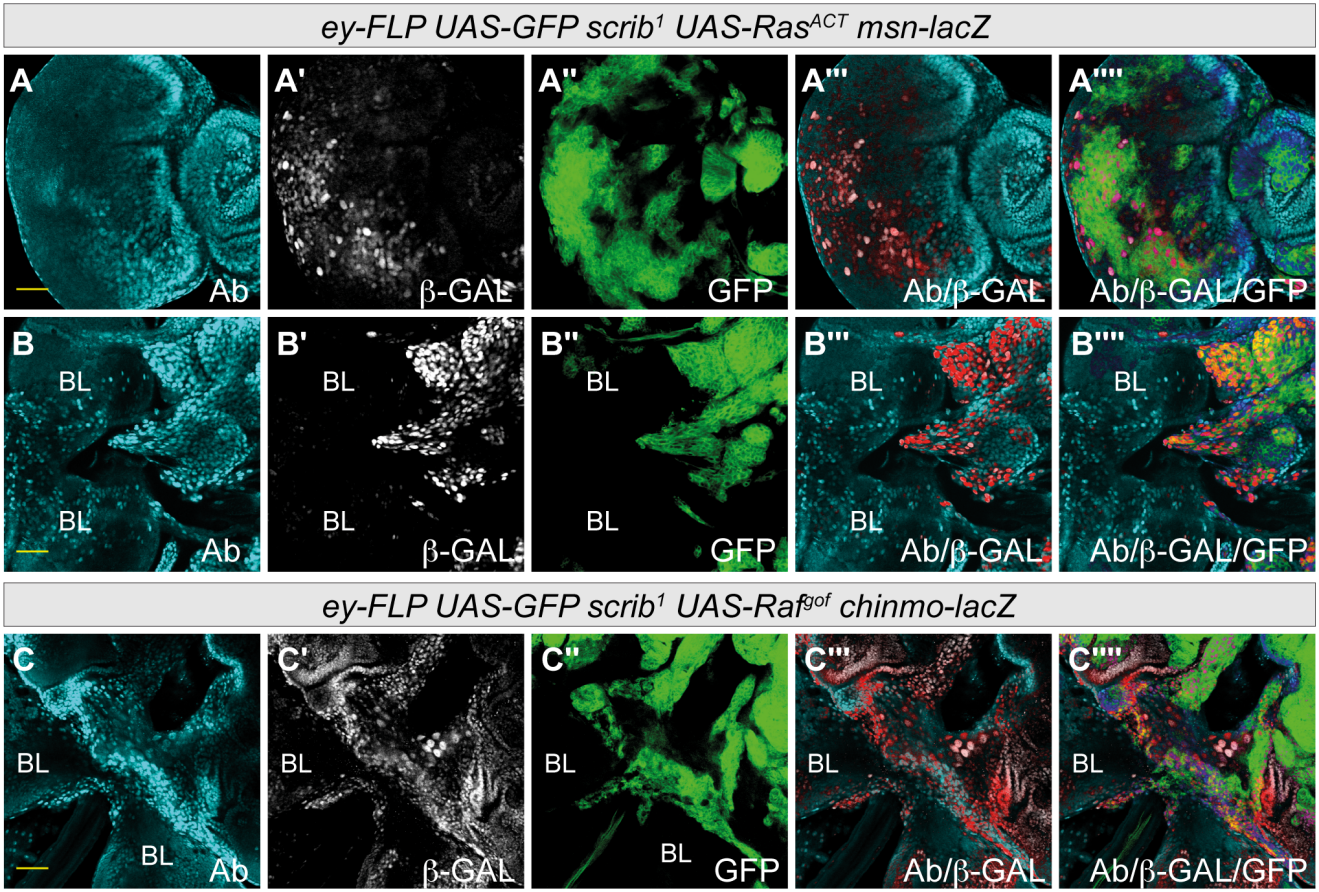
Ab is expressed in *msn-lacZ* and *chinmo-lacZ* expressing tumor cells. Mosaic eye-antennal discs, anterior to the right (A) and attached to brain lobes (BL) (B-C). Clones are generated with *ey-FLP*, and are positively marked by GFP (green, or magenta when overlaid with white). Ab (A-C) expression is shown in cyan (red when overlaid with white, dark blue when overlaid with green or yellow when overlaid with white and green in the merges), *msn-lacZ* (A-B) and *chinmo-lacZ* (C) expression is shown by antibody detection of β-Galactosidase (white, or red when overlaid with cyan in the merges). Yellow scale bar corresponds to 40μm. (A-B) Ab is endogenously expressed in the anterior progenitor domain of the eye disc (A) and is present in basal and migrating cells (B) of *scrib* ^*1*^ *+ Ras* ^*ACT*^ tumors. These same cells strongly express the JNK reporter *msn-lacZ* (A’, B’). See S8A Fig for the endogenous expression of Ab in control eye/antennal discs. (C) The migrating *scrib* ^*1*^ + *Raf* ^*GOF*^ tumor cells express Ab and are also positive for *chinmo-lacZ* expression.

It was possible, however, that a functional role for *chinmo* in tumor formation might be being masked by redundancy with another BTB-ZF protein expressed within the tumors. We therefore turned to the functionally related BTB-ZF protein, Abrupt. Abrupt is also expressed within the eye disc progenitor cells [70, 71], and our own previous work had indicated that Abrupt is highly oncogenic when overexpressed in the *Drosophila* eye-antennal disc [69]. Furthermore, although the transcriptional array did not indicate that *ab* expression was significantly altered by JNK activity within Ras and Notch-driven tumors (S2 Table), Ab protein was present in basal and migrating cells of Ras-driven tumors (Fig 7), and, using the enhancer trap *msn-lacZ* as a reporter for JNK activity, Ab was strongly expressed in *msn-lacZ* positive cells migrating between the brain lobes (Fig 7A and 7B). Immuno-staining to detect *chinmo-lacZ* expression in *scrib* ^-^ + *Raf* ^*gof*^ tumors also showed that Ab protein was present in *chinmo-lacZ* positive cells tumor cells (Fig 7C). However, consistent with the array data, Ab did not appear to be a JNK–induced gene, since Ab levels were not increased in all *msn-lacZ* positive tumor cells, and ectopically activating JNK signaling within eye disc clones by expressing an activated allele of JNKK, *hep* ^*ACT*^, did not lead to increased levels of Ab (S7 Fig). Thus, although Ab is not likely to be a direct target of JNK signaling, it is co-expressed with *chinmo* in JNK-positive cells, and could therefore play a functional role in JNK-driven tumor development.

To analyse the role of *ab* in tumor formation, we used RNAi transgenes to knockdown *ab* in eye disc clones. This showed a strong reduction in Ab protein levels, thereby validating the RNAi lines (S8 Fig). Strikingly, expression of *ab* ^*RNAi*^ in *scrib* ^-^ + *Ras* ^*ACT*^ or *scrib* ^-^ + *N* ^*ACT*^ tumors significantly reduced tumor overgrowth at day 9, thus indicating that Ab was required for tumor development (Fig 6). To next determine if reducing *ab* activity would expose a functional requirement for *chinmo* in tumorigenesis, we coexpressed *ab* ^*RNAi*^ and *chinmo* ^RNAi^ in *scrib* ^-^ + *Ras* ^*ACT*^/*N* ^*ACT*^ tumors. Indeed, this produced a significantly greater reduction to tumor development at day 9, than *ab* ^*RNAi*^ alone, and nearly eliminated tumor overgrowth (Fig 6). Thus, when the activity of Ab, a BTB-ZF protein functionally related to Chinmo, is reduced, a key role for Chinmo in tumor development is exposed.

### *fru* overexpression, but not *br* or *ttk* knockdown, also promotes oncogene-mediated transformation

Our analysis of BTB-ZF proteins expressed in the tumors focussed upon Chinmo and Ab, since evidence suggested that they could be important promoters of a progenitor-like cell state. However, other BTB-ZF genes were also identified in the array as being deregulated by JNK signaling in the tumors. *fru* expression was upregulated by JNK, raising the possibility that *fru*, like *chinmo*, could have oncogenic activity; and *br* and *ttk* were repressed by JNK in the tumors, raising the possibility that they could normally represss tumorigenesis, in a similar way to which some mammalian BTB-ZF proteins are known to function as tumor suppressors.

**Fig 8:**
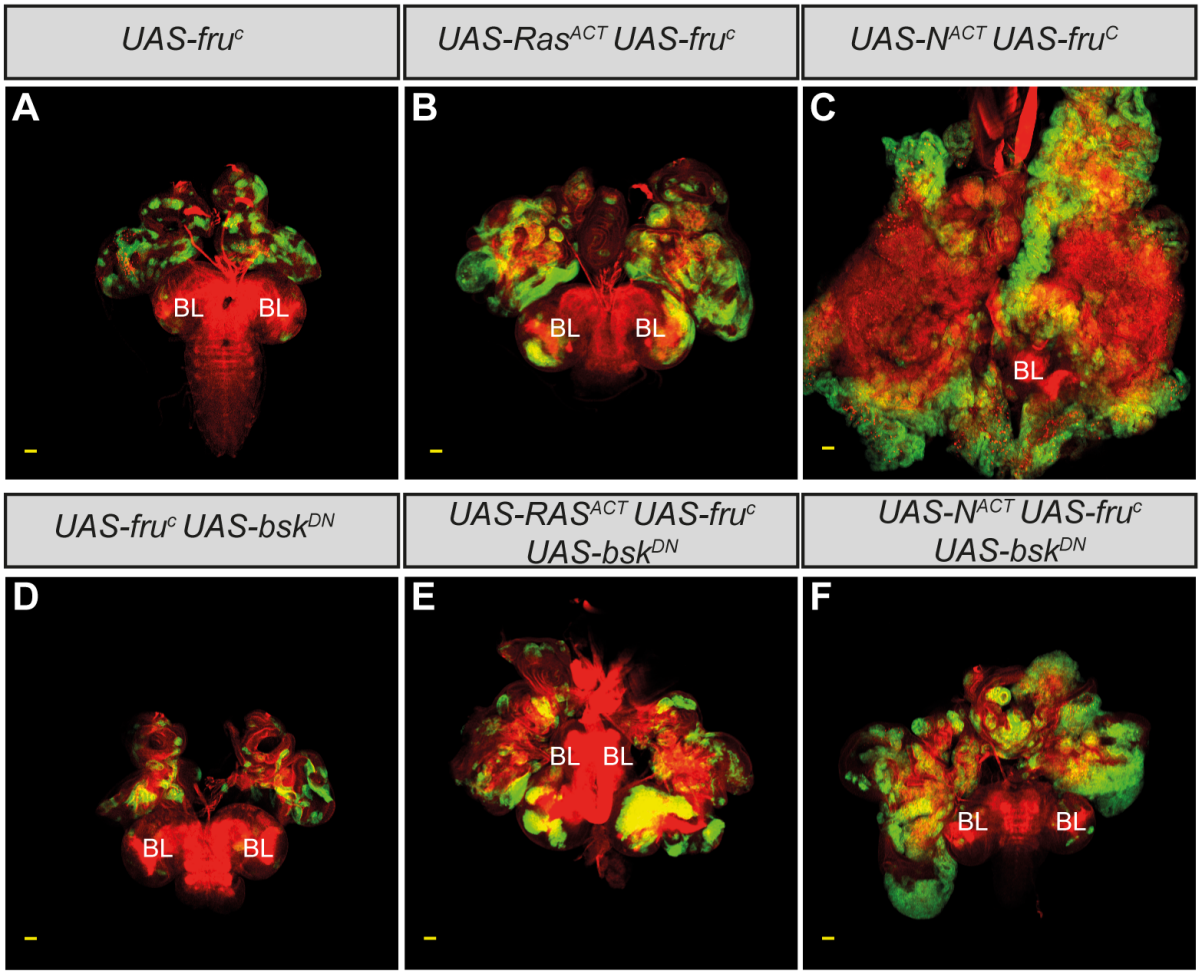
*fru* overexpression cooperates with *Ras* ^*ACT*^ or *N* ^*ACT*^ to promote JNK-independent tumor overgrowth in the eye-antennal disc. Larval mosaic eye-antennal discs attached to brain lobes (BL), anterior to the top, at day 5 (A and D) and day 9 (B, C, E and F). Clones are generated with *ey-FLP*, and are positively marked by GFP (green). Tissue morphology is shown with Phalloidin staining to highlight F-actin (red). The yellow scale bar corresponds to 40μm. (A) Mosaic eye-antennal discs expressing *UAS-fru* ^*C*^ in clones are relatively normal in size (compare to Fig 3A). (B,C) Mosaic eye-antennal discs co-expressing *UAS-fru* ^*C*^ with *UAS-Ras* ^*ACT*^ (B) or *UAS-fru* ^*C*^ with *UAS-N* ^*ACT*^ (C) results in tissue massively overgrowing throughout an extended larval stage of development. (D) Mosaic eye-antennal discs coexpressing *UAS-bsk* ^*DN*^ with *UAS*-*fru* ^*C*^ in clones are similar to *UAS-fru* ^*C*^ clones alone (A). (E-F) Co-expressing *UAS-bsk* ^*DN*^ in *fru* ^*C*^ + *Ras* ^*ACT*^ (E) or *fru* ^*C*^ + *N* ^*ACT*^ (F) clones does not restore pupariation to the tumor-bearing larvae, and the mutant tissue overgrows throughout an extended larval stage of development.

To determine if *fru* could act as an oncogene in *Drosophila*, we overexpressed *fru* in eye-antennal disc clones. The transcriptional regulation of *fru* is complex, involving multiple isoforms of differentially expressed products, however, the overexpression in clones of a *fru* isoform known to be normally expressed in the eye disc [45], did not result in massive clonal overgrowth (Fig 8A), although pupariation was often delayed. In contrast, when ectopic *fru* expression was combined with either *Ras* ^*ACT*^ or *N* ^*ACT*^, massive, but non-invasive, tumor overgrowth ensued during an extended larval stage (Fig 8B and 8C). Similar to *chinmo*-driven tumors, the overgrowth was at least partly JNK-independent, since pupariation was not restored by co-expressing *bsk* ^*DN*^ in *fru* + *Ras* ^*ACT*^ or *fru* + *N* ^*ACT*^ tumors, and tumor overgrowth continued throughout an extended larval stage (Fig 8E and 8F). Thus *fru* over-expression is sufficient to drive cooperative tumorigenesis in the eye-antennal disc, with a similar potency to *chinmo* over-expression.

In contrast to *fru*, *br* and *ttk* were identified in the expression arrays as genes that were repressed by JNK signaling in the tumors. Indeed, immuno-staining of *scrib* ^-^ + *Ras* ^*ACT*^ and *scrib* ^-^ + *N* ^*ACT*^ eye-antennal disc tumors confirmed that Br levels were significantly reduced in the tumors, both within the main tumor mass, as well as in tumor cells migrating between the brain lobes (S9 Fig). To test if the downregulation of either *br* or *ttk* would be sufficient to elicit cooperative tumor overgrowth in combination with oncogenic signals, we knocked down either *br* or *ttk* in clones with RNAi, and co-expressed either *Raf* ^*gof*^ or *N* ^*ACT*^ in the knock-down clones. Interestingly, however, although Br and Ttk protein levels were reduced in clones ectopically expressing *br* ^*RNAi*^ or *ttk* ^*RNAi*^, respectively (S10 Fig), neither *Raf* ^*gof*^ nor *N* ^*ACT*^ was sufficient to elicit *br* ^*RNAi*^ or *ttk* ^*RNAi*^ clonal overgrowth throughout an extended larval stage of development (S10 Fig). Thus, in summary, whilst the overexpression of either *fru* or *chinmo* is sufficient to cooperate with Ras or Notch in *Drosophila* tumorigenesis, the downregulation of BTB-ZF genes does not sensitize cells to Raf or Notch-induced transformation.

## Discussion

In this report we have defined the transcriptional changes induced by JNK signaling within both *scrib* ^-^ + *Ras* ^*ACT*^ and *scrib* ^-^ + *N* ^*ACT*^ tumors by carrying out comparative microarray expression arrays. This showed that JNK exerts a profound effect upon the transcriptional profile of both Ras and Notch-driven tumor types. The expression of nearly 1000 genes was altered by the expression of *bsk* ^*DN*^ in either Ras or Notch-driven tumors, and less than half of these changes were shared between the two tumor types, indicating that JNK signaling elicits unique tumorigenic expression profiles depending upon the cooperating oncogenic signal. Nevertheless, of the 399 JNK-regulated probe sets shared between Ras and Notch-driven tumors, we hypothesized that these had the potential to provide key insights into JNK’s oncogenic activity, and to prioritize these targets, we considered that the expression of the critical oncogenic regulators would not just be altered by *bsk* ^*DN*^, but would be normalized to close to wild type levels. This subset of the 399 probe set was identified by comparing the expression profile of each genotype back to control tissue, thereby producing a more focussed JNK signature of 103 genes. Notably, this included previously characterized targets of JNK in the tumors, such as *Mmp1*, *cher* and *Pax*, thereby providing validation of the approach [16, 21, 23]. Also amongst these candidates were 4 BTB-ZF genes; two of which were upregulated by JNK in the tumors (*chinmo* and *fru*), and two downregulated (*br* and *ttk*). Focussing upon *chinmo*, we showed that *chinmo* overexpression is sufficient to prime epithelial cells for cooperation with *Ras* ^*ACT*^ in both the eye antennal disc and in the adult midgut epithelium, and that *chinmo* is required for cooperative *Ras* ^*ACT*^ or *N* ^*ACT*^-driven tumor overgrowth, although it’s function was only exposed when it’s knockdown was combined with knockdown of a functionally similar BTB-ZF transcription factor, *abrupt*. This family of proteins is highly oncogenic in *Drosophila*, since previous work has shown that *ab* overexpression can cooperate with loss of *scrib* to promote neoplastic overgrowth [69], and in these studies, we also show that overexpression of a *fru* isoform normally expressed in the eye disc is capable of promoting cooperation with Ras^ACT^ and N^ACT^ in the eye-antennal disc, in a similar manner to *chinmo* overexpression. Thus, whether *fru* also plays a role in driving Ras or Notch-driven tumorigenesis warrants further investigation. Indeed, a deeper understanding of the oncogenic activity of these genes is likely to be highly relevant to human tumors, since of the over 40 human BTB-ZF family members, many are implicated in both haematopoietic and epithelial cancers, functioning as either oncogenes (eg. Bcl6, BTB7) or tumor suppressors (eg. PLZF, HIC1) [77]. Furthermore, over-expression of BTB7, can also cooperate with activated Ras in transforming primary cells [78], and its loss makes MEFs refractory to transformation by various key oncogenes such as Myc, H-ras^V12^ and T-Ag [79], suggesting that cooperating mechanisms between BTB-ZF proteins and additional oncogenic stimuli might be conserved.

### The relationship between JNK-induced overgrowth and stemness in *Drosophila* tumors

JNK signaling in *Drosophila* tumors is known to promote tumor overgrowth through both the STAT and Hippo pathways [19, 24–26, 29]. Deregulation of the STAT pathway was evident in the arrays through the upregulation of Upd ligands by JNK in both Ras and Notch-driven tumors. In contrast, although *cher* was identified in the arrays as being upregulated in both tumor types and previous studies have shown that *cher* is partly required for the deregulation of the Hippo pathway in *scrib* ^-^ + *Ras* ^*ACT*^ tumors [23], more direct evidence for Hippo pathway deregulation amongst the JNK signature genes was lacking. In part, this could be due to JNK regulating the pathway through post-transcriptional mechanisms involving direct phosphorylation of pathway components [80]. However, the failure to identify known Hippo pathway target genes, and proliferation response genes in general, may simply highlight limitations in the sensitivity of the array assay and the cut-offs used for determining significance, despite it’s obvious success in correctly identifying many known JNK targets.

Whether tumor overgrowth through STAT and Yki activity is somehow associated with a stem cell or progenitor-like state remains uncertain. Although imaginal discs exhibit developmental plasticity and regeneration potential, and JNK signaling is required for both of these stem-like properties (reviewed in [81]), there is no positive evidence for the existence of a population of asymmetrically dividing stem cells within imaginal discs [82]. Instead, symmetrical divisions of progenitor cells may be the means by which imaginal discs can rapidly generate enough cells to form the differentiated structures of the adult fly. To date, progenitor cells have only been characterized in the eye disc neuroepithelium. These cells have a pseudostratified columnar epithelial morphology and express the MEIS family transcription factor, Hth, which is downregulated as cells initiate differentiation and begin expressing Dac and Eya. Interestingly, they also require Yki for their proliferation [83], and can be induced to overproliferate in response to increased STAT activity [35]. However, analysis of cell fate markers indicated that tumor overgrowth was not likley to be solely due to the overproliferation of these undifferentiated progenitor cells. Although *scrib* ^-^ + *Ras* ^*ACT*^/*N* ^*ACT*^ tumors, were characterized by the failure to transition to Dac/Eya expression in the eye disc, blocking JNK in *scrib* ^-^ + *Ras* ^*ACT*^/*N* ^*ACT*^ tumors did not restore tumor cell differentiation, despite overgrowth being curtailed, and Hth expression was not maintained in the tumors in a JNK-dependent manner. Nevertheless, a JNK-induced gene such as *chinmo* is likely to be associated with promoting a progenitor-like state, since it is a potential STAT target gene required for adult eye development that is expressed in eye disc progenitor cells in response to increased Upd activity [73, 84] and its overexpression alone is sufficient to block Dac/Eya expression. Furthermore, *chinmo* is also required for cyst stem cell maintenance in the *Drosophila* testis [73], and our own work has shown that *chinmo* overexpression promotes increased numbers of *esg>GFP* expressing stem cells or enteroblasts in the adult midgut. As the BTB-ZF protein Ab is also highly oncogenic and expressed in the eye disc progenitor cells, we hypothesize that the JNK-induced expression of *chinmo* in *scrib* ^-^ + *Ras* ^*ACT*^/*N* ^*ACT*^ tumors could cooperate with Ab to maintain a progenitor-like cell state in the eye disc, and that this is required for *scrib* ^-^ + *Ras* ^*ACT*^/*N* ^*ACT*^ tumor overgrowth. However, although Ab was expressed in *chinmo-*expressing, JNK positive tumor cells, *Ab* does not appear to be a JNK-induced gene. What JNK-independent mechanisms control *ab* expression will therefore require further analysis. Interestingly, we have previously observed that *ab* overexpression in eye disc clones upregulates *chinmo* expression [69] and although the effect of *chinmo* expression upon *ab* is yet to be described, the data at least suggest that the control of their expression is interlinked in a yet to be defined manner.

Consistent with Chinmo being important for *scrib* ^-^ + *Ras* ^*ACT*^/*N* ^*ACT*^ tumor overgrowth, *chinmo* overexpression itself is also highly oncogenic. Over-expression of *chinmo* with *Ras* ^*ACT*^ or *N* ^*ACT*^ drives tumorigenesis in the eye-antennal disc, and also resulted in enlarged brain lobes, presumably due to the generation of overexpressing clones within the neuroepithelium of the optic lobes. In the adult midgut, the overexpression of *chinmo* with *Ras* ^*ACT*^ in the stem cell and its immediate progeny, the enteroblast, promoted massive tumor overgrowth, resulting in *esg<GFP* expressing cells completely filling the lumen of the gut, and eventual host lethality. The luminal filling of *esg<GFP* cells is reminiscent of the effects of *Ras* ^*ACT*^ expression in larval adult midgut progenitor cells [74]. Together with the data linking Chinmo function to stem or progenitor cells, these data reinforce the idea that epithelial tumorigenesis can be primed by signals, such as *chinmo* over-expression, that promote a stem or progenitor cell state.

The function of some *Drosophila* BTB-ZF proteins including Chinmo and Ab, has also been linked to heterochronic roles involving the conserved *let-7* miRNA pathway and hormone signals, to regulate the timing of differentiation [38, 70–72, 85]. Indeed, Ab can directly bind to the steroid hormone receptor co-activator Taiman (Tai or AIB1/SRC3 in humans), to represses the transcriptional response to ecdysone signaling [85]. Thus, the capacity of BTB-ZF proteins to influence the timing of developmental transitions, particularly if they impede developmental transitions within stem or progenitor cells, could help account for their potent oncogenic activity. Indeed, ecdysone-response genes were repressed by JNK in the tumorigenic state, consistent with the failure of the larvae to pupate and a delay in developmental timing. Whether repressing the ecdysone response cell autonomously might contribute to tumor overgrowth and/or invasion will be an interesting area of future investigation, given the complex role of hormone signaling in mammalian stem cell biology and cancers.

### The relationship between JNK-induced invasion and progenitor states

Previous studies have suggested that JNK-dependent tumor cell invasion is developmentally similar to the JNK-induced EMT-like events occurring during imaginal disc eversion [32]. Thus the capacity of JNK to also promote tumor overgrowth is reminiscent of how EMT inducers such as Twist (Twi) and Snail (Sna) are associated with the acquisition of cancer stem cell properties [86]. In *Drosophila*, however, *twi* and *sna* were not induced by JNK in the tumors, although transcription factors involved in mesoderm specification, including the NF-κB homologue, *dl* (a member of the 103 JNK signature), and *Mef2* (a member of the 399 JNK signature), were amongst the up-regulated JNK targets. Mesoderm specification is not necessarily associated with a mesenchymal-like cell morphology, however, *dl* is involved in the induction of EMT during embryonic development, and both dl and Mef2 act with Twi and Sna to coordinate mesoderm formation [63]. Interestingly, we recently identified *dl* in an overexpression screen for genes capable of cooperating with *scrib* ^-^ in *Drosophila* tumorigenesis [69], and Mef2 has been identified as a cooperating oncogene in *Drosophila*, and possibly also in humans, where a correlation exists between the expression of Notch and Mef2 paralogues in human breast tumor samples [87]. It is therefore possible that dl and Mef2 either act in combination with Twi or Sna, or independently of them but in a similar oncogenic capacity, to promote a mesodermal cell fate in *scrib* ^-^ + *Ras* ^*ACT*^/*N* ^*ACT*^ tumors. The potential relevance of this to the mesenchymal cell morphology associated with tumor cell invasion, as well as the acquisition of progenitor states is worthy of further investigation.

In *mef2*-driven tumors both overgrowth and invasion depend upon activation of JNK signaling [87], suggesting that Mef2 is not capable of promoting invasive capabilities independent of JNK. In contrast, *chinmo* + *Ras* ^*ACT*^/*N* ^*ACT*^ tumors appeared non-invasive and retained epithelial morphology despite the massive overgrowth, although closer examination of cell polarity markers will be required to confirm this. Furthermore, the overgrowth of *chinmo* + *Ras* ^*ACT*^/*N* ^*ACT*^ tumors was not dependent upon JNK signaling, suggesting that the maintenance of a progenitor-like state could be uncoupled from JNK-induced EMT-effectors associated with invasion. Whether clear divisions between mesenchymal behaviour and progenitor states in tumors can be clearly separated in this manner is not yet clear, however, overall, it is likely that multiple JNK-regulated genes will participate in both promoting tumor overgrowth as well as migration/invasion. Although we used the 103 JNK signature as a means to focus upon potential key candidates, an analysis of the 399 JNK-regulated probe sets common to both Ras and Notch-driven tumours has the potential to provide deeper insights into the multiple effectors of JNK signaling during tumorigenesis. Whilst the individual role of these genes can be probed with knockdowns, the complexity of the response, potentially with multiple redundancies and cross-talk, will ultimately need a network level of understanding to more fully expose key nodes participating in overgrowth and invasion. This approach has considerable potential to further expose core principles and mechanisms that drive human tumorigenesis, since it is clear that many fundamental commonalities underlie the development of tumors in *Drosophila* and mammals.

## Supporting Information

S1 Fig Blocking JNK signaling in *scrib* ^*1*^ + *Ras* ^*ACT*^ tumors restores Eya, but not Dac or Ato, expression. Mosaic eye-antennal discs, anterior to the right. Clones are generated with *ey-FLP*, and are positively marked by GFP (green, or magenta when overlaid with white). Cell fate is marked by the expression of Ato, Eya and Dac (white, or magenta when overlaid with GFP in the merges). Yellow scale bar corresponds to 40μM. (A-D) Control *FRT82B* (A) and *UAS-Ras* ^*ACT*^ (B) eye-antennal discs show the normal pattern of Ato expression, however, Ato levels are downregulated in *scrib* ^-^ + *Ras* ^*ACT*^ tumors (C, arrowhead), and remain repressed in *scrib* ^*1*^ + *Ras* ^*ACT*^ + *bsk* ^*DN*^ clones (D, arrowhead). (E-H) Eya is expressed in the anterior portion of the eye disc in control *FRT82B* discs (E) and is ectopically expressed in *UAS-Ras* ^*ACT*^ clones (F), but it is repressed in *scrib* ^*1*^ + *Ras* ^*ACT*^ tumors (G, arrowhead). In *scrib* ^*1*^ + *Ras* ^*ACT*^ + *bsk* ^*DN*^ clones, Eya expression is restored and upregulated (H, arrowhead). (I-L) Dac is expressed in a band of cells extending across the eye disc in control *FRT82B* discs (I), and is downregulated in *UAS-Ras* ^*ACT*^ clones (J) and *scrib* ^*1*^ + *Ras* ^*ACT*^ tumors (K, arrowhead). Blocking JNK in *scrib* ^*1*^ + *Ras* ^*ACT*^ tumors does not restore Dac expression (L, arrowhead).
(TIF)

S2 Fig Blocking JNK signaling in *scrib* ^*1*^ + *N* ^*ACT*^ tumors does not restore Ato, Eya or Dac expression to the tumor cells. Mosaic eye/antennal discs, anterior to the right. Clones are generated with *ey-FLP*, and are positively marked by GFP (green, or magenta when overlaid with white). Cell fate is marked by the expression of Ato, Eya and Dac (white, or magenta when overlaid with GFP in the merges). Yellow scale bar corresponds to 40μM. (A-C) Ato levels are downregulated in *scrib* ^*1*^ + *N* ^*ACT*^ tumors (B, arrowhead), and remain repressed in *scrib* ^*1*^ + *N* ^*ACT*^ + *bsk* ^*DN*^ clones (C, arrowhead). (D-F) Eya is downregulated in *scrib* ^*1*^ + *N* ^*ACT*^ tumors (E, arrowhead), and in *scrib* ^*1*^ + *N* ^*ACT*^ + *bsk* ^DN^ clones (F, arrowhead). (G-I) Dac is downregulated in *scrib* ^*1*^ + *N* ^*ACT*^ tumors (H, arrowhead), and in *scrib* ^*1*^ + *N* ^*ACT*^ + *bsk* ^DN^ clones (I, arrowhead).
(TIF)

S3 Fig Hth is not required for *scrib* ^*1*^ + *N* ^*ACT*^ tumor overgrowth. Mosaic eye-antennal discs, anterior to the right. Brain lobes (BL) are also shown in (F). Clones are generated with *ey-FLP*, and are positively marked by GFP (green, or magenta when overlaid with white). Hth or Elav is white, or magenta when overlaid with GFP in the merges. Yellow scale bar corresponds to 40μM. (A-D) In control *FRT82B* eye-antennal mosaic discs, Hth is expressed in the antennal disc, the progenitor domain of the eye disc, and in the posterior of the eye disc (A, arrowheads). In *scrib* ^*1*^ + *Ras* ^*ACT*^ tumors, Hth levels are reduced in all three regions (B). In contrast, *scrib* ^*1*^ + *N* ^*ACT*^ tumors maintain Hth expression throughout the eye disc and show mild ectopic expression (C, arrowheads), this ectopic expression is maintained in *scrib* ^*1*^ + *N* ^*ACT*^ + *bsk* ^*DN*^ clones (D, arrowhead). (E-F) Expressing *UAS-N* ^*ACT*^ in *scrib* ^*1*^ *hth* ^*P2*^ double mutant clones results in large clones (E) and does not abrogate tumor development throughout an extended larval stage (compare F to control *FRT82B* mosaic eye-antennal discs attached to brain lobes in Fig 3A).
(TIF)

S4 Fig The brain lobes in *chinmo* ^*FL*^ + *Ras* ^*ACT*^ tumor-bearing larvae massively overgrow. Mosaic eye-antennal discs, anterior to the top, attached to brain lobes (BL). Clones are generated with *ey-FLP*, and are positively marked by GFP (green, or magenta when overlaid with white). Tissue morphology is shown with Phalloidin staining to highlight F-actin (white, or magenta when overlaid with GFP). Yellow scale bar corresponds to 40μM. (A,B) In control *FRT82B* mosaic larvae, small GFP-positive clones of tissue are visible in the brain lobes (A). In *chinmo* ^*FL*^ + *Ras* ^*ACT*^ tumor-bearing larvae, the brain lobes are massively enlarged and predominantly consist of GFP-positive tumor tissue (B’).
(TIF)

S5 Fig Quantification of *esg>GFP* cells and Prospero-positive cells in adult midguts overexpressing *chinmo* ^*FL*^. The number of total cells, prospero-positive cells, and GFP-positive cells were calculated from confocal sections of adult midguts expressing either *UAS-GFP* or *UAS-GFP* + *UAS-chinmo* ^*FL*^, under the control of *esg-GAL4*,*tub-GAL80* ^*ts*^, for 5 days (control) or 10 days at 29°C. The overexpression of *chinmo* significantly increases the number of GFP-positive cells. Error bars are the mean with 95% CI. n = 13 (control), 7 (chinmo day 5), 6 (chinmo day 10), and refers to the number of sections analysed, with each section being from a different fly.
(TIF)

S6 Fig Knockdown of *chinmo* in the wing disc reduces the levels of Chinmo protein. Larval wing discs, of *ptc-GAL4* day 5 larvae expressing *UAS-GFP* (green) and *UAS*-*chinmo* ^*RNAi*^ (white, or magenta when overlaid with GFP). (A-C) In control wing discs *UAS-GFP* is expressed along the anterior-posterior boundary by *ptc-GAL4* and Chinmo expression is ubiquitous across the disc (A). Chinmo levels are decreased in the *ptc-GAL4* domain when the *UAS-chinmo* ^*RNAi-17156R-2*^ transgene is expressed (B). The fold change in Chinmo expression is quantified (C), error bars are SD, n = 3, ** p<0.01.
(TIF)

S7 Fig Ab levels are not increased in clones with ectopically activated JNK signaling. Mosaic eye-antennal discs, anterior to the right. Clones are generated with *ey-FLP*, and are positively marked by GFP (green, or magenta when overlaid with white). Ab is detected by immunohistochemical staining (white, or magenta when overlaid with GFP in the merges). (A) The expression of an activated allele of JNKK (*UAS-hemipterous(hep)* ^*ACT*^) in eye-antennal disc clones produces very small clones due to cell death, but the co-expression of the caspase inhibitor *UAS-P35* permits the analysis of larger clones of tissue. Ab is normally expressed in the anterior progenitor domain of the eye disc and in the antennal disc, and its levels are not increased in *hep* ^*ACT*^ + *P35* clones.
(TIF)

S8 Fig Knockdown of *ab* reduces the levels of Ab protein. Mosaic eye-antennal discs, anterior to the right. Clones are generated with *ey-FLP*, and are positively marked by GFP (green, or magenta when overlaid with white). Ab is detected by immunohistochemical staining (white, or magenta when overlaid with GFP in the merges). Yellow scale bar corresponds to 40μM. (A-C) Control FRT82B mosaic discs show the endogenous expression of Ab in the eye progenitor domain (A, arrowhead) and antennal disc. *UAS*-*ab* ^*RNAi#104582*^ expressing clones show decreased Ab protein levels (B, arrowheads). *UAS-ab* ^*RNAi#4807R-2*^ expressing clones also show decreased Ab protein levels (C, arrowheads).
(TIF)

S9 Fig Br is repressed within Ras and Notch-driven tumors, and within tumor cells migrating between the brain lobes. Mosaic eye-antennal discs, anterior to the right. Brain lobes (BL) are also shown in (D). Clones are generated with *ey-FLP*, and are positively marked by GFP (green, or magenta when overlaid with white). Br is detected by immunohistochemical staining (white, or magenta when overlaid with GFP in the merges). Tissue morphology is shown with phalloidin staining F-actin in (D, red). Yellow scale bar corresponds to 40μM. (A-D) In control *FRT82B* mosaic discs, Broad is expressed in both the eye and antennal disc (A). In *scrib* ^*1*^ + *N* ^*ACT*^ (B, arrowhead) and *scrib* ^*1*^ + Ras^*ACT*^ tumors (C, arrowhead), Br levels are reduced. *scrib* ^*1*^ + *Ras* ^*ACT*^ tumor cells, which are known to be active for JNK-pathway activity [16], migrate between the brain lobes and do not express Br (D).
(TIF)

S10 Fig Knockdown of *br* or *ttk* in eye-antennal disc clones is not sufficient to promote cooperation with *Raf* ^*gof*^ or *N* ^*ACT*^ in tumorigenesis. Mosaic eye-antennal discs, anterior to the right. Clones are generated with *ey-FLP*, and are positively marked by GFP (green, or magenta when overlaid with white). Br, Ttk and Elav are detected by immunohistochemical staining (white, or magenta when overlaid with GFP in the merges). Yellow scale bar corresponds to 40μM. (A-C) *UAS-br* ^*RNAi#104648*^ expressing clones show decreased Br protein levels (A, arrowheads). Co-expressing *UAS-br* ^*RNAi#104648*^ in *UAS-Raf* ^*GOF*^ (B) and *UAS-N* ^*ACT*^ (C) clones does not result in tumorigenesis. (D-F) Ttk levels are reduced in *UAS-ttk* ^*RNAi#101980*^ expressing clones (D, arrowheads), and co-expression of *UAS-ttk* ^*RNAi#101980*^ in *UAS-Raf* ^*GOF*^ (E) and *UAS-N* ^*ACT*^ (F) clones does not result in tumorigenesis.
(TIF)

S1 File List of differentially expressed probes (log base 2 fold change>1 and p<0.05) in *scrib* ^*1*^ + *Ras* ^*ACT*^ mosaic eye-antennal discs compared to *scrib* ^*1*^ + *Ras* ^*ACT*^ + *bsk* ^*DN*^ discs, and *scrib* ^*1*^ + *N* ^*ACT*^ mosaic eye-antennal discs compared to *scrib* ^*1*^ + *N* ^*ACT*^ + *bsk* ^*DN*^ discs (see Fig 1C). Of the 1463 probes deregulated in both comparisons, 429 probes (pattern 2) are unique to *scrib* ^*1*^ + *Ras* ^*ACT*^ versus *scrib* ^*1*^ + *Ras* ^*ACT*^ + *bsk* ^*DN*^ discs, 635 probes (pattern 1) are unique to *scrib* ^*1*^ + *N* ^*ACT*^ versus *scrib* ^*1*^ + *N* ^*ACT*^ + *bsk* ^*DN*^ discs, and 399 probes (pattern 3) are common to both *scrib* ^*1*^ + *Ras* ^*ACT*^ versus *scrib* ^*1*^ + *Ras* ^*ACT*^ + *bsk* ^*DN*^ and *scrib* ^*1*^ + *N* ^*ACT*^ versus *scrib* ^*1*^ + *N* ^*ACT*^ + *bsk* ^*DN*^ discs. 17489 probes were not significantly deregulated in either comparison.
(CSV)

S2 File List of differentially expressed probes (log base 2 fold change>1 and p<0.05) in *scrib* ^*1*^ + *Ras* ^*ACT*^, *scrib* ^*1*^ + *N* ^*ACT*^, *scrib* ^*1*^ + *Ras* ^*ACT*^ + *bsk* ^*DN*^ and *scrib* ^*1*^ + *N* ^*ACT*^ + *bsk* ^*DN*^ mosaic eye-antennal discs compared to control *FRT82B* mosaic eye-antennal discs (see Fig 1E). Of the 2117 deregulated probes, 168 probes (pattern 12) were deregulated in both *scrib* ^*1*^ + *Ras* ^*ACT*^ and *scrib* ^*1*^ + *N* ^*ACT*^ mosaic eye-antennal discs (compared to the *FRT82B* control), but not in *scrib* ^*1*^ + *Ras* ^*ACT*^ + *bsk* ^*DN*^ and *scrib* ^*1*^ + *N* ^*ACT*^ + *bsk* ^*DN*^ mosaic eye-antennal discs (compared to the *FRT82B* control), and are considered to represent a JNK-signature.
(CSV)

S1 Table Expression of candidate genes in *scrib* ^-^ + *Ras* ^*ACT*^ and *scrib* ^-^ + *N* ^*ACT*^ tumors (+/- *bsk* ^*DN*^) compared to control *FRT82B* eye-antennal discs, and in *scrib* ^-^ + *Ras* ^*ACT*^ and *scrib* ^-^ + *N* ^*ACT*^ tumors compared to their respective genotypes expressing *bsk* ^*DN*^. (DOC)

S2 Table Expression of BTB-ZF genes in *scrib* ^-^ + *Ras* ^*ACT*^ and *scrib* ^-^ + *N* ^*ACT*^ tumors (+/- *bsk* ^*DN*^) compared to control *FRT82B* eye-antennal discs. (DOC)

## References

[1] Kalluri R, Weinberg RA. The basics of epithelial-mesenchymal transition. J Clin Invest. 2009;119(6):1420–8. 10.1172/JCI39104 19487818 PMC2689101

[2] Thiery JP, Acloque H, Huang RY, Nieto MA. Epithelial-mesenchymal transitions in development and disease. Cell. 2009;139(5):871–90. 10.1016/j.cell.2009.11.007 19945376

[3] Polyak K, Weinberg RA. Transitions between epithelial and mesenchymal states: acquisition of malignant and stem cell traits. Nat Rev Cancer. 2009;9(4):265–73. 10.1038/nrc2620 19262571

[4] Lopez-Novoa JM, Nieto MA. Inflammation and EMT: an alliance towards organ fibrosis and cancer progression. EMBO Mol Med. 2009;1(6–7):303–14. 10.1002/emmm.200900043 20049734 PMC3378143

[5] Iliopoulos D, Hirsch HA, Struhl K. An epigenetic switch involving NF-kappaB, Lin28, Let-7 MicroRNA, and IL6 links inflammation to cell transformation. Cell. 2009;139(4):693–706. 10.1016/j.cell.2009.10.014 19878981 PMC2783826

[6] Rhim AD, Mirek ET, Aiello NM, Maitra A, Bailey JM, McAllister F, et al EMT and Dissemination Precede Pancreatic Tumor Formation. Cell. 2012;148(1–2):349–61. 10.1016/j.cell.2011.11.025 22265420 PMC3266542

[7] Xie G, Yao Q, Liu Y, Du S, Liu A, Guo Z, et al IL-6-induced epithelial-mesenchymal transition promotes the generation cultures. Int J Oncol. 2011 . 22134360 10.3892/ijo.2011.1275 PMC3584811

[8] Asiedu MK, Ingle JN, Behrens MD, Radisky DC, Knutson KL. TGFbeta/TNF(alpha)-mediated epithelial-mesenchymal transition generates breast cancer stem cells with a claudin-low phenotype. Cancer Res. 2011;71(13):4707–19. 10.1158/0008-5472.CAN-10-4554 21555371 PMC3129359

[9] Min C, Eddy SF, Sherr DH, Sonenshein GE. NF-kappaB and epithelial to mesenchymal transition of cancer. J Cell Biochem. 2008;104(3):733–44. 10.1002/jcb.21695 18253935

[10] Peinado H, Olmeda D, Cano A. Snail, Zeb and bHLH factors in tumour progression: an alliance against the epithelial phenotype? Nat Rev Cancer. 2007;7(6):415–28. . 17508028 10.1038/nrc2131

[11] Wu KJ, Yang MH. Epithelial-mesenchymal transition and cancer stemness: the Twist1-Bmi1 connection. Biosci Rep. 2011;31(6):449–55. 10.1042/BSR20100114 21919891

[12] Cordenonsi M, Zanconato F, Azzolin L, Forcato M, Rosato A, Frasson C, et al The Hippo transducer TAZ confers cancer stem cell-related traits on breast cancer cells. Cell. 2011;147(4):759–72. 10.1016/j.cell.2011.09.048 22078877

[13] Brumby AM, Richardson HE. Using Drosophila melanogaster to map human cancer pathways. Nat Rev Cancer. 2005;5(8):626–39. . 16034367 10.1038/nrc1671

[14] Brumby AM, Richardson HE. scribble mutants cooperate with oncogenic Ras or Notch to cause neoplastic overgrowth in Drosophila. Embo J. 2003;22(21):5769–79. . 14592975 10.1093/emboj/cdg548 PMC275405

[15] Pagliarini RA, Xu T. A genetic screen in Drosophila for metastatic behavior. Science. 2003;302(5648):1227–31. . 14551319 10.1126/science.1088474

[16] Leong GR, Goulding KR, Amin N, Richardson HE, Brumby AM. Scribble mutants promote aPKC and JNK-dependent epithelial neoplasia independently of Crumbs. BMC Biol. 2009;7:62 10.1186/1741-7007-7-62 19778415 PMC2760524

[17] Cordero JB, Macagno JP, Stefanatos RK, Strathdee KE, Cagan RL, Vidal M. Oncogenic Ras diverts a host TNF tumor suppressor activity into tumor promoter. Dev Cell. 2010;18(6):999–1011. 10.1016/j.devcel.2010.05.014 20627081 PMC3175220

[18] Igaki T, Pastor-Pareja JC, Aonuma H, Miura M, Xu T. Intrinsic tumor suppression and epithelial maintenance by endocytic activation of Eiger/TNF signaling in Drosophila. Dev Cell. 2009;16(3):458–65. 10.1016/j.devcel.2009.01.002 19289090 PMC2729686

[19] Pastor-Pareja JC, Wu M, Xu T. An innate immune response of blood cells to tumors and tissue damage in Drosophila. Dis Model Mech. 2008;1(2–3):144–54; discussion 53. 10.1242/dmm.000950 19048077 PMC2562178

[20] Igaki T, Pagliarini RA, Xu T. Loss of cell polarity drives tumor growth and invasion through JNK activation in Drosophila. Curr Biol. 2006;16(11):1139–46. . 16753569 10.1016/j.cub.2006.04.042

[21] Uhlirova M, Bohmann D. JNK- and Fos-regulated Mmp1 expression cooperates with Ras to induce invasive tumors in Drosophila. Embo J. 2006;25(22):5294–304. . 17082773 10.1038/sj.emboj.7601401 PMC1636619

[22] Brumby AM, Goulding KR, Schlosser T, Loi S, Galea R, Khoo P, et al Identification of Novel Ras-cooperating Oncogenes in Drosophila melanogaster: A RhoGEF/Rho-family/JNK Pathway is a Central Driver of Tumorigenesis. Genetics. 2011 . 21368274 10.1534/genetics.111.127910 PMC3120157

[23] Kulshammer E, Uhlirova M. The actin cross-linker Filamin/Cheerio mediates tumor malignancy downstream of JNK signaling. J Cell Sci. 2012 . 23239028 10.1242/jcs.114462

[24] Bunker BD, Nellimoottil TT, Boileau RM, Classen AK, Bilder D. The transcriptional response to tumorigenic polarity loss in Drosophila. Elife. 2015;4 Epub 2015/02/27. 10.7554/eLife.03189 25719210 PMC4369581

[25] Wu M, Pastor-Pareja JC, Xu T. Interaction between Ras(V12) and scribbled clones induces tumour growth and invasion. Nature. 2010;463(7280):545–8. 10.1038/nature08702 20072127 PMC2835536

[26] Davie K, Jacobs J, Atkins M, Potier D, Christiaens V, Halder G, et al Discovery of transcription factors and regulatory regions driving in vivo tumor development by ATAC-seq and FAIRE-seq open chromatin profiling. PLoS Genet. 2015;11(2):e1004994 Epub 2015/02/14. 10.1371/journal.pgen.1004994PGENETICS-D-14-02754 [pii]. 25679813 PMC4334524

[27] Perez-Garijo A, Shlevkov E, Morata G. The role of Dpp and Wg in compensatory proliferation and in the formation of hyperplastic overgrowths caused by apoptotic cells in the Drosophila wing disc. Development. 2009;136(7):1169–77. 10.1242/dev.034017 19244279

[28] Sun G, Irvine KD. Regulation of Hippo signaling by Jun kinase signaling during compensatory cell proliferation and regeneration, and in neoplastic tumors. Dev Biol. 2011;350(1):139–51. 10.1016/j.ydbio.2010.11.036 21145886 PMC3038240

[29] Doggett K, Grusche FA, Richardson HE, Brumby AM. Loss of the Drosophila cell polarity regulator Scribbled promotes epithelial tissue overgrowth and cooperation with oncogenic Ras-Raf through impaired Hippo pathway signaling. BMC Dev Biol. 2011;11(1):57 . 21955824 10.1186/1471-213X-11-57 PMC3206446

[30] Colombani J, Andersen DS, Leopold P. Secreted peptide Dilp8 coordinates Drosophila tissue growth with developmental timing. Science. 2012;336(6081):582–5. 10.1126/science.1216689 22556251

[31] Garelli A, Gontijo AM, Miguela V, Caparros E, Dominguez M. Imaginal discs secrete insulin-like peptide 8 to mediate plasticity of growth and maturation. Science. 2012;336(6081):579–82. 10.1126/science.1216735 22556250

[32] Srivastava A, Pastor-Pareja JC, Igaki T, Pagliarini R, Xu T. Basement membrane remodeling is essential for Drosophila disc eversion and tumor invasion. Proc Natl Acad Sci U S A. 2007;104(8):2721–6. . 17301221 10.1073/pnas.0611666104 PMC1815248

[33] Biteau B, Hochmuth CE, Jasper H. JNK activity in somatic stem cells causes loss of tissue homeostasis in the aging Drosophila gut. Cell Stem Cell. 2008;3(4):442–55. 10.1016/j.stem.2008.07.024 18940735 PMC3225008

[34] Jiang H, Patel PH, Kohlmaier A, Grenley MO, McEwen DG, Edgar BA. Cytokine/Jak/Stat signaling mediates regeneration and homeostasis in the Drosophila midgut. Cell. 2009;137(7):1343–55. 10.1016/j.cell.2009.05.014 19563763 PMC2753793

[35] Tsai YC, Sun YH. Long-range effect of upd, a ligand for Jak/STAT pathway, on cell cycle in Drosophila eye development. Genesis. 2004;39(2):141–53. . 15170700 10.1002/gene.20035

[36] Lee JD, Treisman JE. The role of Wingless signaling in establishing the anteroposterior and dorsoventral axes of the eye disc. Development. 2001;128(9):1519–29. . 11290291 10.1242/dev.128.9.1519

[37] Adachi-Yamada T, Nakamura M, Irie K, Tomoyasu Y, Sano Y, Mori E, et al p38 mitogen-activated protein kinase can be involved in transforming growth factor beta superfamily signal transduction in Drosophila wing morphogenesis. Mol Cell Biol. 1999;19(3):2322–9. . 10022918 10.1128/mcb.19.3.2322 PMC84024

[38] Zhu S, Lin S, Kao CF, Awasaki T, Chiang AS, Lee T. Gradients of the Drosophila Chinmo BTB-zinc finger protein govern neuronal temporal identity. Cell. 2006;127(2):409–22. . 17055440 10.1016/j.cell.2006.08.045

[39] Rieckhof GE, Casares F, Ryoo HD, Abu-Shaar M, Mann RS. Nuclear translocation of extradenticle requires homothorax, which encodes an extradenticle-related homeodomain protein. Cell. 1997;91(2):171–83. . 9346235 10.1016/s0092-8674(00)80400-6

[40] Spradling AC, Stern D, Beaton A, Rhem EJ, Laverty T, Mozden N, et al The Berkeley Drosophila Genome Project gene disruption project: Single P-element insertions mutating 25% of vital Drosophila genes. Genetics. 1999;153(1):135–77. . 10471706 10.1093/genetics/153.1.135 PMC1460730

[41] Go MJ, Eastman DS, Artavanis-Tsakonas S. Cell proliferation control by Notch signaling in Drosophila development. Development. 1998;125(11):2031–40. . 9570768 10.1242/dev.125.11.2031

[42] Brand AH, Perrimon N. Raf acts downstream of the EGF receptor to determine dorsoventral polarity during Drosophila oogenesis. Genes Dev. 1994;8(5):629–39. . 7926754 10.1101/gad.8.5.629

[43] Karim FD, Rubin GM. Ectopic expression of activated Ras1 induces hyperplastic growth and increased cell death in Drosophila imaginal tissues. Development. 1998;125(1):1–9. . 9389658 10.1242/dev.125.1.1

[44] Bilder D, Perrimon N. Localization of apical epithelial determinants by the basolateral PDZ protein Scribble. Nature. 2000;403(6770):676–80. . 10688207 10.1038/35001108

[45] Song HJ, Billeter JC, Reynaud E, Carlo T, Spana EP, Perrimon N, et al The fruitless gene is required for the proper formation of axonal tracts in the embryonic central nervous system of Drosophila. Genetics. 2002;162(4):1703–24. Epub 2003/01/14. 12524343 10.1093/genetics/162.4.1703 PMC1462372

[46] Hinz U, Giebel B, Campos-Ortega JA. The basic-helix-loop-helix domain of Drosophila lethal of scute protein is sufficient for proneural function and activates neurogenic genes. Cell. 1994;76(1):77–87. Epub 1994/01/14. doi: 0092-8674(94)90174-0 [pii]. . 8287481 10.1016/0092-8674(94)90174-0

[47] Lee T, Luo L. Mosaic analysis with a repressible cell marker for studies of gene function in neuronal morphogenesis. Neuron. 1999;22(3):451–61. . 10197526 10.1016/s0896-6273(00)80701-1

[48] Jarman AP, Sun Y, Jan LY, Jan YN. Role of the proneural gene, atonal, in formation of Drosophila chordotonal organs and photoreceptors. Development. 1995;121(7):2019–30. . 7635049 10.1242/dev.121.7.2019

[49] Ryoo HD, Mann RS. The control of trunk Hox specificity and activity by Extradenticle. Genes Dev. 1999;13(13):1704–16. . 10398683 10.1101/gad.13.13.1704 PMC316852

[50] Team RC. R: A language and environment for statistical computing. R Foundation for Statistical Computing, Vienna, Austria 2014 Available from: http://www.R-project.org/.

[51] Gentleman RC, Carey VJ, Bates DM, Bolstad B, Dettling M, Dudoit S, et al Bioconductor: open software development for computational biology and bioinformatics. Genome Biol. 2004;5(10):R80. Epub 2004/10/06. doi: gb-2004-5-10-r80 [pii] 10.1186/gb-2004-5-10-r80 15461798 PMC545600

[52] Gautier L, Cope L, Bolstad BM, Irizarry RA. affy—analysis of Affymetrix GeneChip data at the probe level. Bioinformatics. 2004;20(3):307–15. Epub 2004/02/13. 10.1093/bioinformatics/btg405 20/3/307 [pii]. . 14960456

[53] Wu JaI, R with contributions from MacDonald, J. and Gentry, J. gcrma: Background Adjustment Using Sequence Information. R package version 2.38.0.

[54] Bolstad B. Low Level Analysis of High-density Oligonucleotide Array Data: Background, Normalization and Summarization: University of California, Berkeley; 2004.

[55] Smyth GK. Limma: linear models for microarray data In: Gentleman R, Carey VJ, Huber W, Irizarry RA, Dudoit S, editors. Bioinformatics and Computational Biology Solutions using R and Bioconductor Statistics for Biology and Health. New York: Springer; 2005 p. pp 397–420.

[56] Benjamini Y, and Hochberg Y. Controlling the false discovery rate: a practical and powerful approach to multiple testing. Journal of the Royal Statistical Society Series. 1995;B(57):289–300.

[57] Subramanian A, Tamayo P, Mootha VK, Mukherjee S, Ebert BL, Gillette MA, et al Gene set enrichment analysis: a knowledge-based approach for interpreting genome-wide expression profiles. Proc Natl Acad Sci U S A. 2005;102(43):15545–50. Epub 2005/10/04. doi: 0506580102 [pii] 10.1073/pnas.0506580102 16199517 PMC1239896

[58] Carbon S, Ireland A, Mungall CJ, Shu S, Marshall B, Lewis S. AmiGO: online access to ontology and annotation data. Bioinformatics. 2009;25(2):288–9. Epub 2008/11/27. doi: btn615 [pii] 10.1093/bioinformatics/btn615 19033274 PMC2639003

[59] Martin-Blanco E, Gampel A, Ring J, Virdee K, Kirov N, Tolkovsky AM, et al puckered encodes a phosphatase that mediates a feedback loop regulating JNK activity during dorsal closure in Drosophila. Genes Dev. 1998;12(4):557–70. Epub 1998/03/21. 9472024 10.1101/gad.12.4.557 PMC316530

[60] Rousset R, Bono-Lauriol S, Gettings M, Suzanne M, Speder P, Noselli S. The Drosophila serine protease homologue Scarface regulates JNK signalling in a negative-feedback loop during epithelial morphogenesis. Development. 2010;137(13):2177–86. Epub 2010/06/10. doi: 137/13/2177 [pii] 10.1242/dev.050781 . 20530545

[61] Ohsawa S, Sugimura K, Takino K, Xu T, Miyawaki A, Igaki T. Elimination of oncogenic neighbors by JNK-mediated engulfment in Drosophila. Dev Cell. 2011;20(3):315–28. 10.1016/j.devcel.2011.02.007 21397843

[62] Bond D, Foley E. A quantitative RNAi screen for JNK modifiers identifies Pvr as a novel regulator of Drosophila immune signaling. PLoS Pathog. 2009;5(11):e1000655 10.1371/journal.ppat.1000655 19893628 PMC2766254

[63] Sandmann T, Girardot C, Brehme M, Tongprasit W, Stolc V, Furlong EE. A core transcriptional network for early mesoderm development in Drosophila melanogaster. Genes Dev. 2007;21(4):436–49. . 17322403 10.1101/gad.1509007 PMC1804332

[64] Campbell K, Whissell G, Franch-Marro X, Batlle E, Casanova J. Specific GATA factors act as conserved inducers of an endodermal-EMT. Dev Cell. 2011;21(6):1051–61. 10.1016/j.devcel.2011.10.005 22172671

[65] VanZomeren-Dohm A, Sarro J, Flannery E, Duman-Scheel M. The Drosophila Netrin receptor frazzled/DCC functions as an invasive tumor suppressor. BMC Dev Biol. 2011;11:41 10.1186/1471-213X-11-41 21672235 PMC3144007

[66] Manhire-Heath R, Golenkina S, Saint R, Murray MJ. Netrin-dependent downregulation of Frazzled/DCC is required for the dissociation of the peripodial epithelium in Drosophila. Nat Commun. 2013;4:2790 10.1038/ncomms3790 24225841

[67] Kumar JP. Retinal determination the beginning of eye development. Curr Top Dev Biol. 2010;93:1–28. 10.1016/B978-0-12-385044-7.00001-1 20959161 PMC5830122

[68] Jasper H, Benes V, Schwager C, Sauer S, Clauder-Munster S, Ansorge W, et al The genomic response of the Drosophila embryo to JNK signaling. Dev Cell. 2001;1(4):579–86. Epub 2001/11/13. doi: S1534-5807(01)00045-4 [pii]. . 11703947 10.1016/s1534-5807(01)00045-4

[69] Turkel N, Sahota VK, Bolden JE, Goulding KR, Doggett K, Willoughby LF, et al The BTB-zinc finger transcription factor abrupt acts as an epithelial oncogene in Drosophila melanogaster through maintaining a progenitor-like cell state. PLoS Genet. 2013;9(7):e1003627 10.1371/journal.pgen.1003627 23874226 PMC3715428

[70] Wu YC, Chen CH, Mercer A, Sokol NS. Let-7-complex microRNAs regulate the temporal identity of Drosophila mushroom body neurons via chinmo. Dev Cell. 2012;23(1):202–9. 10.1016/j.devcel.2012.05.013 22814608 PMC3401410

[71] Kucherenko MM, Barth J, Fiala A, Shcherbata HR. Steroid-induced microRNA let-7 acts as a spatio-temporal code for neuronal cell fate in the developing Drosophila brain. Embo J. 2012;31(24):4511–23. 10.1038/emboj.2012.298 23160410 PMC3545287

[72] Caygill EE, Johnston LA. Temporal regulation of metamorphic processes in Drosophila by the let-7 and miR-125 heterochronic microRNAs. Curr Biol. 2008;18(13):943–50. 10.1016/j.cub.2008.06.020 18571409 PMC2736146

[73] Flaherty MS, Salis P, Evans CJ, Ekas LA, Marouf A, Zavadil J, et al chinmo is a functional effector of the JAK/STAT pathway that regulates eye development, tumor formation, and stem cell self-renewal in Drosophila. Dev Cell. 2010;18(4):556–68. 10.1016/j.devcel.2010.02.006 20412771 PMC2859208

[74] Jiang H, Edgar BA. EGFR signaling regulates the proliferation of Drosophila adult midgut progenitors. Development. 2009;136(3):483–93. 10.1242/dev.026955 19141677 PMC2687592

[75] Micchelli CA, Perrimon N. Evidence that stem cells reside in the adult Drosophila midgut epithelium. Nature. 2006;439(7075):475–9. . 16340959 10.1038/nature04371

[76] Apidianakis Y, Pitsouli C, Perrimon N, Rahme L. Synergy between bacterial infection and genetic predisposition in intestinal dysplasia. Proc Natl Acad Sci U S A. 2009;106(49):20883–8. Epub 2009/11/26. doi: 0911797106 [pii] 10.1073/pnas.0911797106 19934041 PMC2791635

[77] Costoya JA. Functional analysis of the role of POK transcriptional repressors. Brief Funct Genomic Proteomic. 2007;6(1):8–18. . 17384421 10.1093/bfgp/elm002

[78] Shvarts A, Brummelkamp TR, Scheeren F, Koh E, Daley GQ, Spits H, et al A senescence rescue screen identifies BCL6 as an inhibitor of anti-proliferative p19(ARF)-p53 signaling. Genes Dev. 2002;16(6):681–6. . 11914273 10.1101/gad.929302 PMC155362

[79] Maeda T, Hobbs RM, Merghoub T, Guernah I, Zelent A, Cordon-Cardo C, et al Role of the proto-oncogene Pokemon in cellular transformation and ARF repression. Nature. 2005;433(7023):278–85. . 15662416 10.1038/nature03203

[80] Sun G, Irvine KD. Ajuba family proteins link JNK to Hippo signaling. Sci Signal. 2013;6(292):ra81. Epub 2013/09/12. doi: 6/292/ra81 [pii] 10.1126/scisignal.2004324 24023255 PMC3830546

[81] Worley MI, Setiawan L, Hariharan IK. Regeneration and transdetermination in Drosophila imaginal discs. Annu Rev Genet. 2012;46:289–310. 10.1146/annurev-genet-110711-155637 22934642

[82] Sustar A, Bonvin M, Schubiger M, Schubiger G. Drosophila twin spot clones reveal cell division dynamics in regenerating imaginal discs. Dev Biol. 2011;356(2):576–87. 10.1016/j.ydbio.2011.06.018 21722631 PMC3144724

[83] Peng HW, Slattery M, Mann RS. Transcription factor choice in the Hippo signaling pathway: homothorax and yorkie regulation of the microRNA bantam in the progenitor domain of the Drosophila eye imaginal disc. Genes Dev. 2009;23(19):2307–19. 10.1101/gad.1820009 19762509 PMC2758742

[84] Flaherty MS, Zavadil J, Ekas LA, Bach EA. Genome-wide expression profiling in the Drosophila eye reveals unexpected repression of notch signaling by the JAK/STAT pathway. Dev Dyn. 2009;238(9):2235–53. 10.1002/dvdy.21989 19504457 PMC2846647

[85] Jang AC, Chang YC, Bai J, Montell D. Border-cell migration requires integration of spatial and temporal signals by the BTB protein Abrupt. Nat Cell Biol. 2009;11(5):569–79. 10.1038/ncb1863 19350016 PMC2675665

[86] Mani SA, Guo W, Liao MJ, Eaton EN, Ayyanan A, Zhou AY, et al The epithelial-mesenchymal transition generates cells with properties of stem cells. Cell. 2008;133(4):704–15. Epub 2008/05/20. doi: S0092-8674(08)00444-3 [pii] 10.1016/j.cell.2008.03.027 18485877 PMC2728032

[87] Pallavi SK, Ho DM, Hicks C, Miele L, Artavanis-Tsakonas S. Notch and Mef2 synergize to promote proliferation and metastasis through JNK signal activation in Drosophila. Embo J. 2012;31(13):2895–907. 10.1038/emboj.2012.129 22580825 PMC3395089

