# Supplementary figures and images for: BTB-Zinc Finger Oncogenes Are Required for Ras and Notch-Driven Tumorigenesis in Drosophila
Source: PLoS One. 2015 Jul 24;10(7):e0132987. doi: 10.1371/journal.pone.0132987 (PMC4514741; doi:10.1371/journal.pone.0132987)

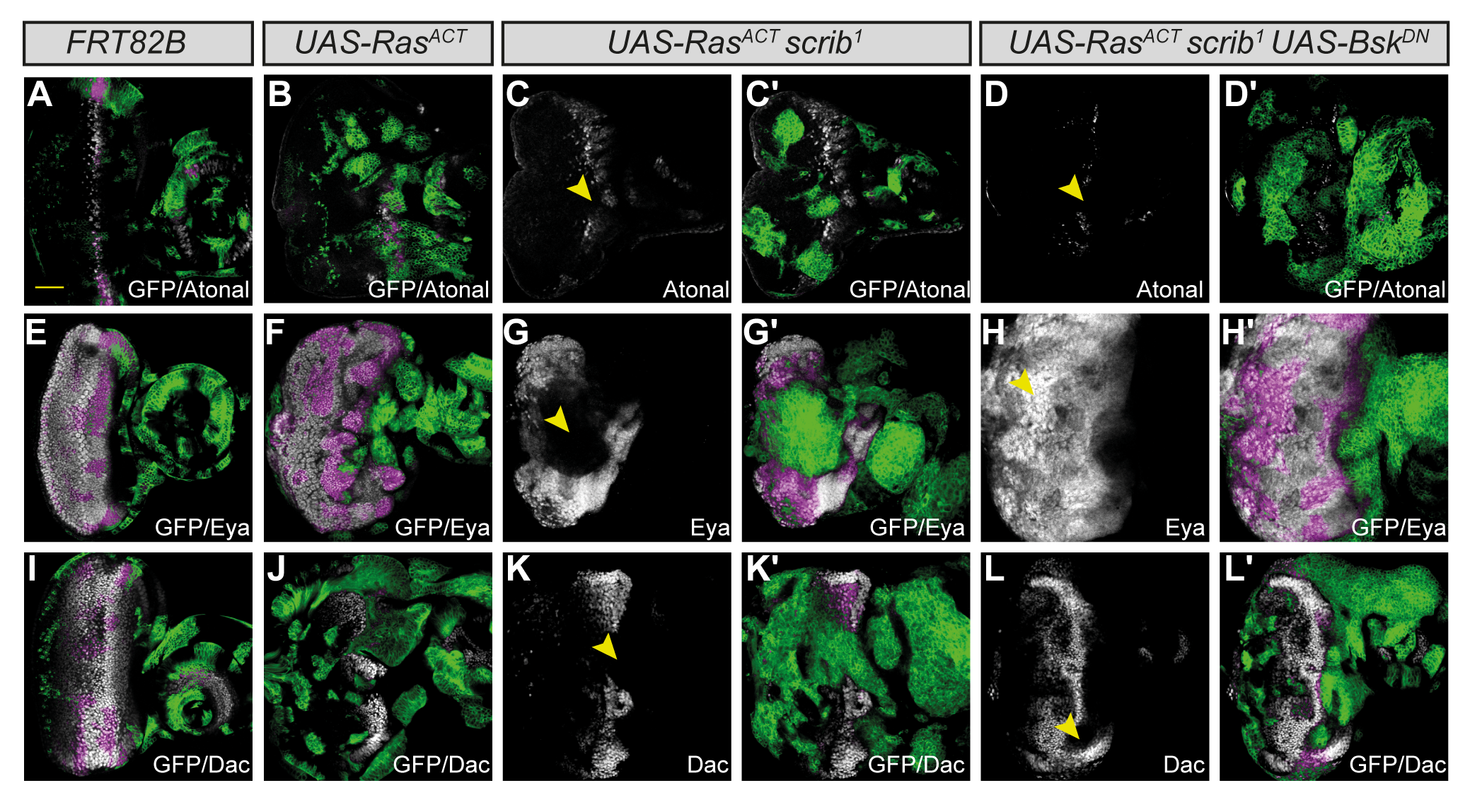

Supplement: S1 Fig — Mosaic eye-antennal discs, anterior to the right. Clones are generated with ey-FLP, and are positively marked by GFP (green, or magenta when overlaid with white). Cell fate is marked by the expression of Ato, Eya and Dac (white, or magenta when overlaid with GFP in the merges). Yellow scale bar corresponds to 40μM. (A-D) Control FRT82B (A) and UAS-Ras ACT (B) eye-antennal discs show the normal pattern of Ato expression, however, Ato levels are downregulated in scrib - + Ras ACT tumors (C, arrowhead), and remain repressed in scrib 1 + Ras ACT + bsk DN clones (D, arrowhead). (E-H) Eya is expressed in the anterior portion of the eye disc in control FRT82B discs (E) and is ectopically expressed in UAS-Ras ACT clones (F), but it is repressed in scrib 1 + Ras ACT tumors (G, arrowhead). In scrib 1 + Ras ACT + bsk DN clones, Eya expression is restored and upregulated (H, arrowhead). (I-L) Dac is expressed in a band of cells extending across the eye disc in control FRT82B discs (I), and is downregulated in UAS-Ras ACT clones (J) and scrib 1 + Ras ACT tumors (K, arrowhead). Blocking JNK in scrib 1 + Ras ACT tumors does not restore Dac expression (L, arrowhead). (TIF) [file pone.0132987.s001.tif]

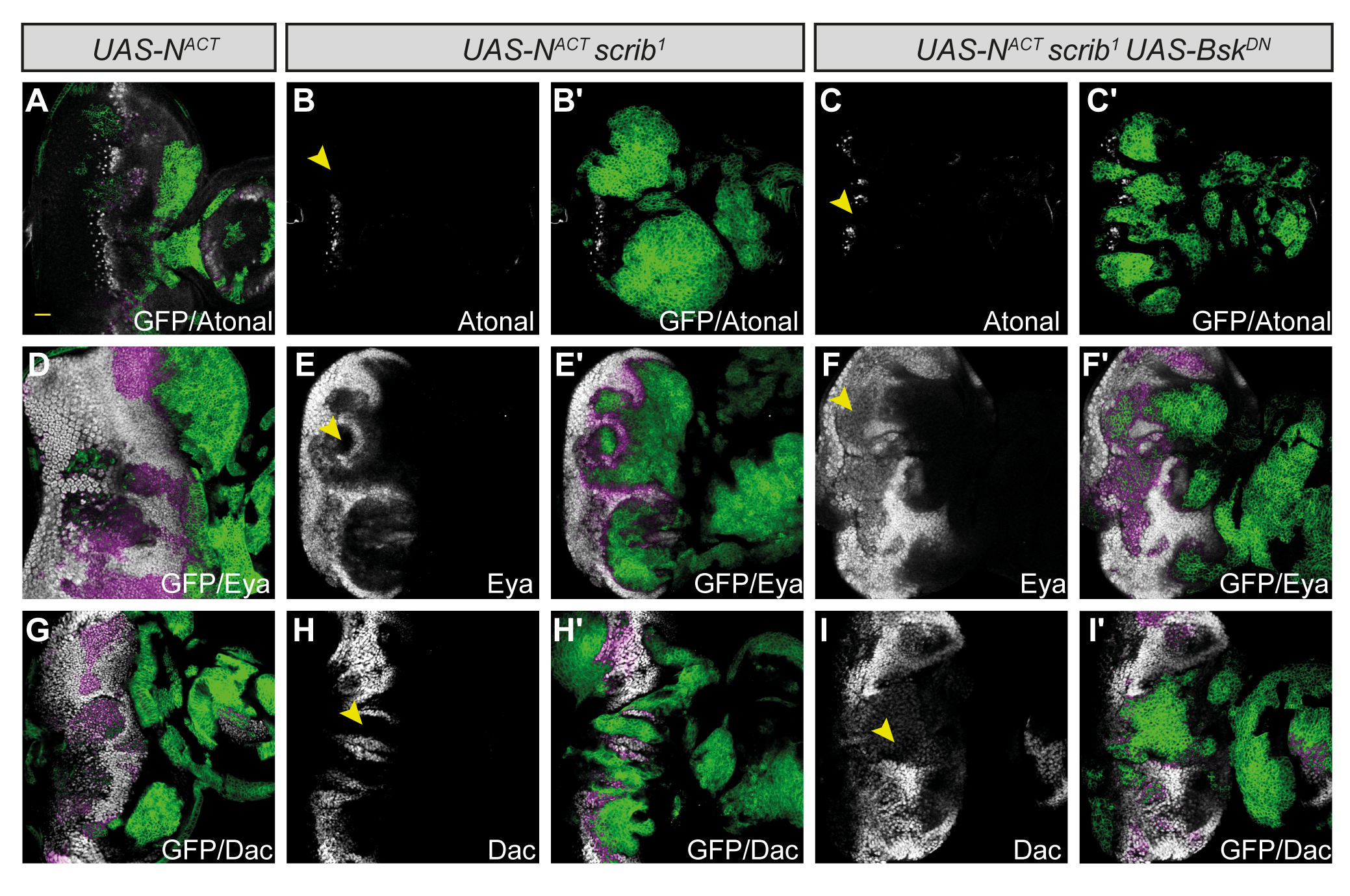

Supplement: S2 Fig — Mosaic eye/antennal discs, anterior to the right. Clones are generated with ey-FLP, and are positively marked by GFP (green, or magenta when overlaid with white). Cell fate is marked by the expression of Ato, Eya and Dac (white, or magenta when overlaid with GFP in the merges). Yellow scale bar corresponds to 40μM. (A-C) Ato levels are downregulated in scrib 1 + N ACT tumors (B, arrowhead), and remain repressed in scrib 1 + N ACT + bsk DN clones (C, arrowhead). (D-F) Eya is downregulated in scrib 1 + N ACT tumors (E, arrowhead), and in scrib 1 + N ACT + bsk DN clones (F, arrowhead). (G-I) Dac is downregulated in scrib 1 + N ACT tumors (H, arrowhead), and in scrib 1 + N ACT + bsk DN clones (I, arrowhead). (TIF) [file pone.0132987.s002.tif]

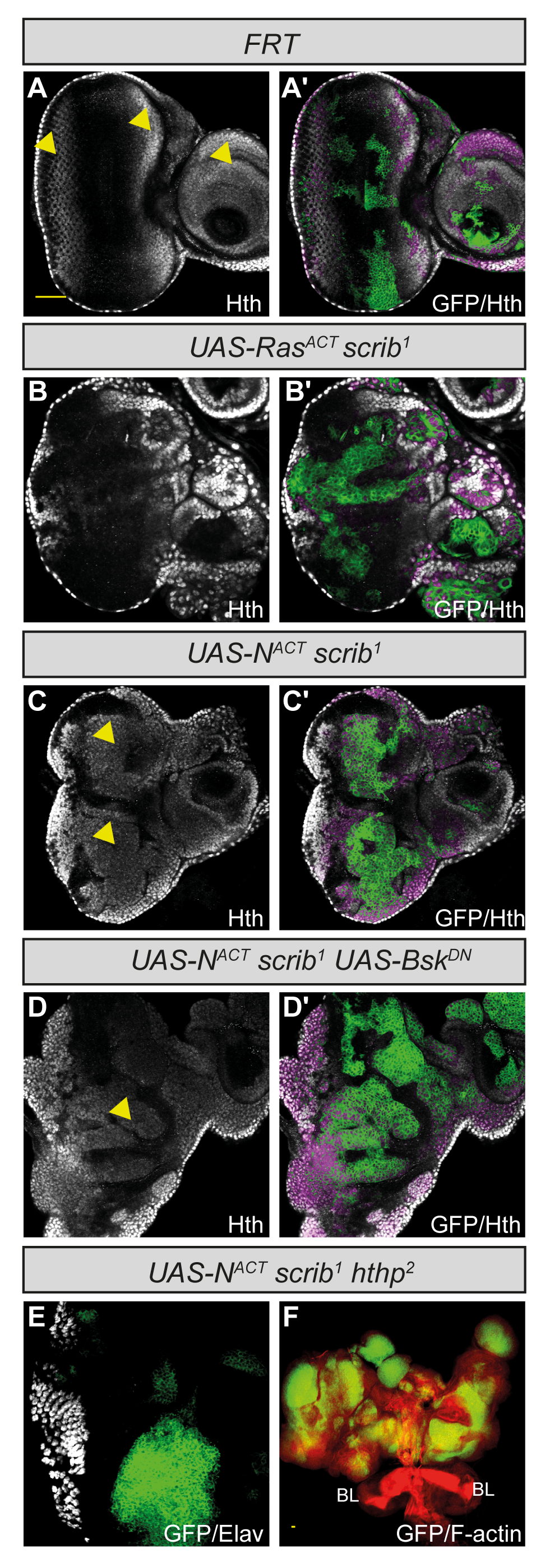

Supplement: S3 Fig — Mosaic eye-antennal discs, anterior to the right. Brain lobes (BL) are also shown in (F). Clones are generated with ey-FLP, and are positively marked by GFP (green, or magenta when overlaid with white). Hth or Elav is white, or magenta when overlaid with GFP in the merges. Yellow scale bar corresponds to 40μM. (A-D) In control FRT82B eye-antennal mosaic discs, Hth is expressed in the antennal disc, the progenitor domain of the eye disc, and in the posterior of the eye disc (A, arrowheads). In scrib 1 + Ras ACT tumors, Hth levels are reduced in all three regions (B). In contrast, scrib 1 + N ACT tumors maintain Hth expression throughout the eye disc and show mild ectopic expression (C, arrowheads), this ectopic expression is maintained in scrib 1 + N ACT + bsk DN clones (D, arrowhead). (E-F) Expressing UAS-N ACT in scrib 1 hth P2 double mutant clones results in large clones (E) and does not abrogate tumor development throughout an extended larval stage (compare F to control FRT82B mosaic eye-antennal discs attached to brain lobes in Fig 3A). (TIF) [file pone.0132987.s003.tif]

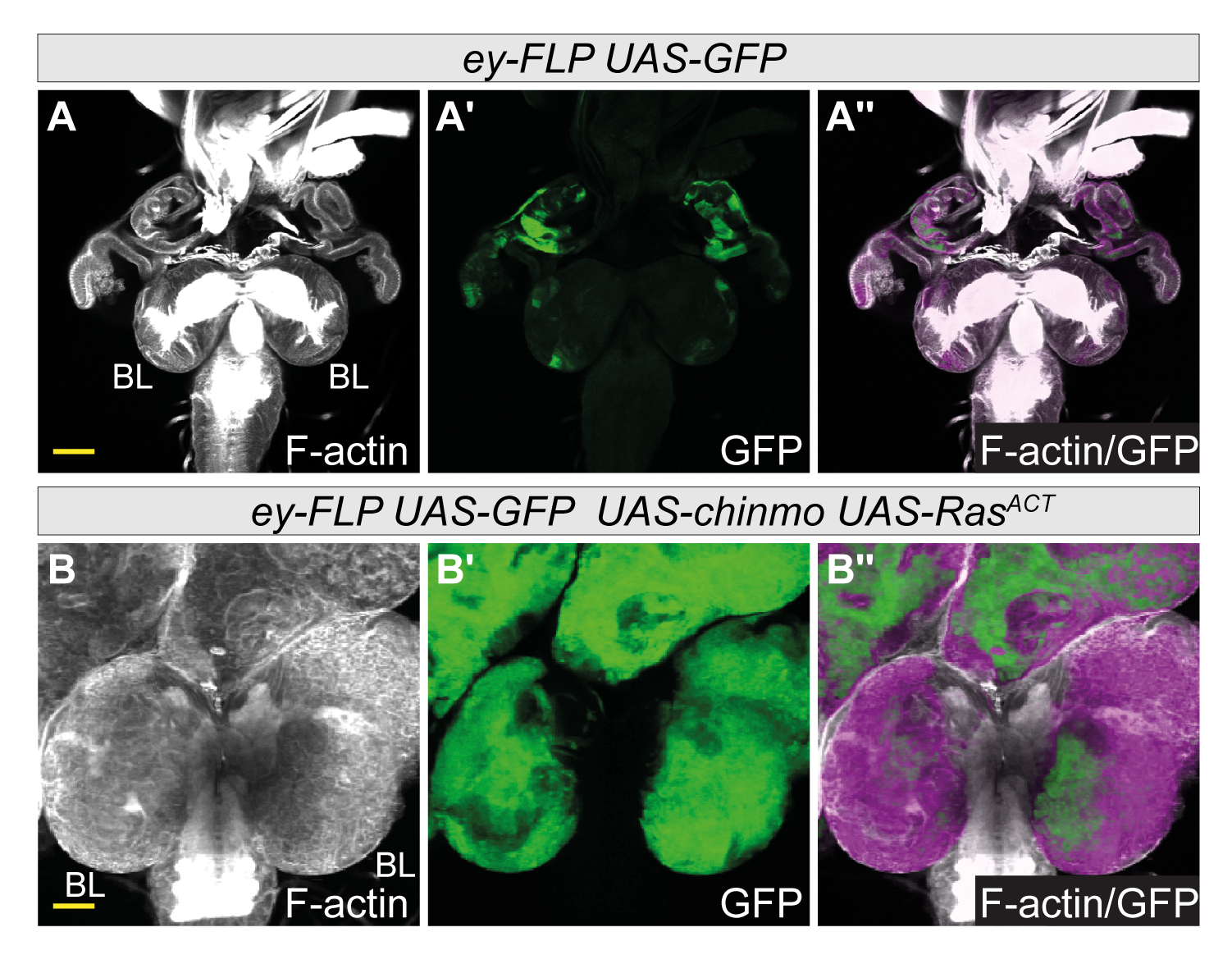

Supplement: S4 Fig — Mosaic eye-antennal discs, anterior to the top, attached to brain lobes (BL). Clones are generated with ey-FLP, and are positively marked by GFP (green, or magenta when overlaid with white). Tissue morphology is shown with Phalloidin staining to highlight F-actin (white, or magenta when overlaid with GFP). Yellow scale bar corresponds to 40μM. (A,B) In control FRT82B mosaic larvae, small GFP-positive clones of tissue are visible in the brain lobes (A). In chinmo FL + Ras ACT tumor-bearing larvae, the brain lobes are massively enlarged and predominantly consist of GFP-positive tumor tissue (B’). (TIF) [file pone.0132987.s004.tif]

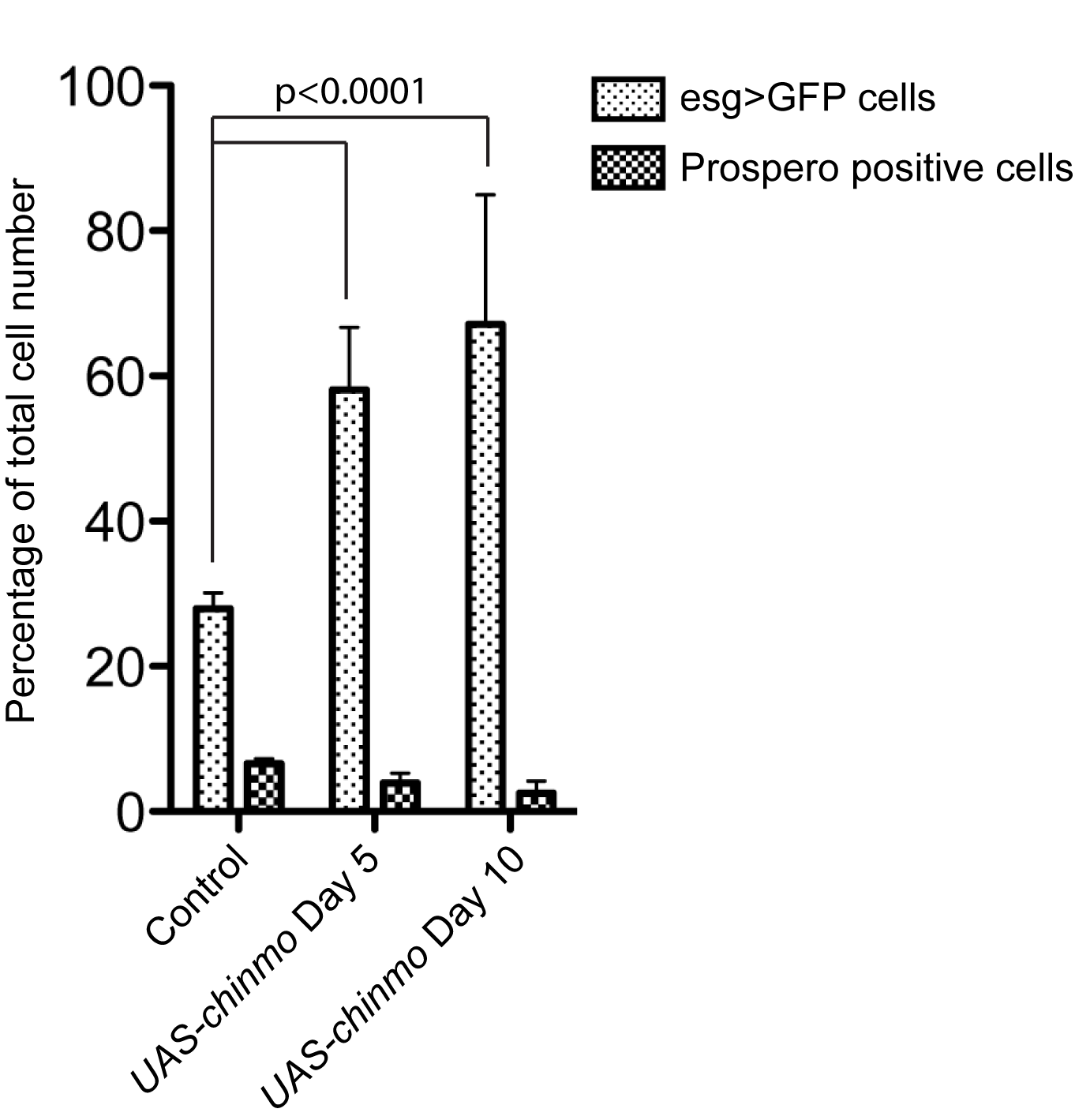

Supplement: S5 Fig — The number of total cells, prospero-positive cells, and GFP-positive cells were calculated from confocal sections of adult midguts expressing either UAS-GFP or UAS-GFP + UAS-chinmo FL, under the control of esg-GAL4,tub-GAL80 ts, for 5 days (control) or 10 days at 29°C. The overexpression of chinmo significantly increases the number of GFP-positive cells. Error bars are the mean with 95% CI. n = 13 (control), 7 (chinmo day 5), 6 (chinmo day 10), and refers to the number of sections analysed, with each section being from a different fly. (TIF) [file pone.0132987.s005.tif]

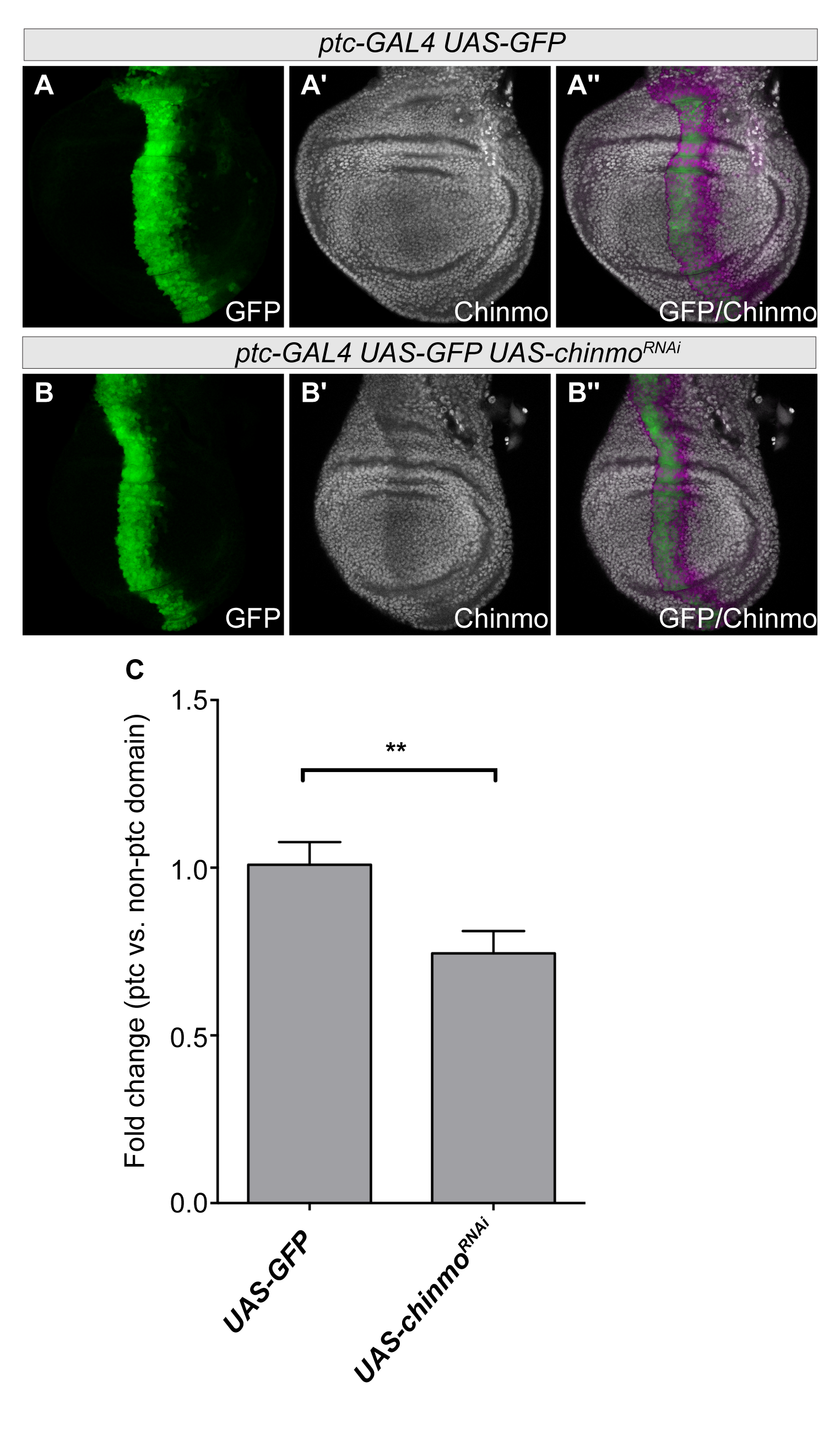

Supplement: S6 Fig — Larval wing discs, of ptc-GAL4 day 5 larvae expressing UAS-GFP (green) and UAS-chinmo RNAi (white, or magenta when overlaid with GFP). (A-C) In control wing discs UAS-GFP is expressed along the anterior-posterior boundary by ptc-GAL4 and Chinmo expression is ubiquitous across the disc (A). Chinmo levels are decreased in the ptc-GAL4 domain when the UAS-chinmo RNAi-17156R-2 transgene is expressed (B). The fold change in Chinmo expression is quantified (C), error bars are SD, n = 3, ** p<0.01. (TIF) [file pone.0132987.s006.tif]

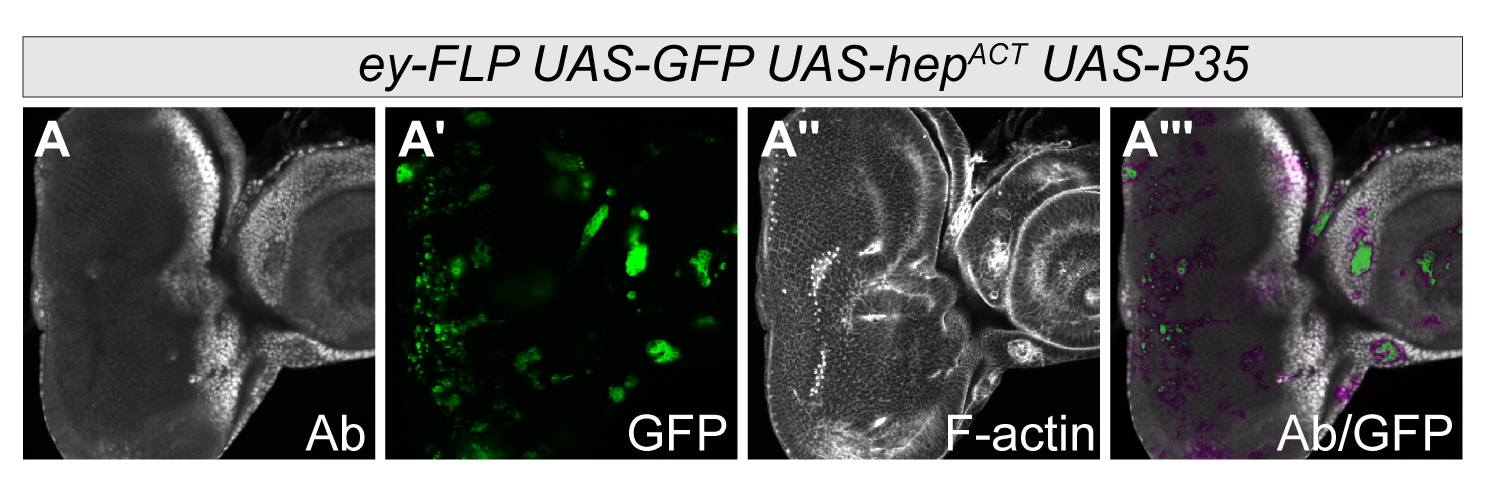

Supplement: S7 Fig — Mosaic eye-antennal discs, anterior to the right. Clones are generated with ey-FLP, and are positively marked by GFP (green, or magenta when overlaid with white). Ab is detected by immunohistochemical staining (white, or magenta when overlaid with GFP in the merges). (A) The expression of an activated allele of JNKK (UAS-hemipterous(hep) ACT) in eye-antennal disc clones produces very small clones due to cell death, but the co-expression of the caspase inhibitor UAS-P35 permits the analysis of larger clones of tissue. Ab is normally expressed in the anterior progenitor domain of the eye disc and in the antennal disc, and its levels are not increased in hep ACT + P35 clones. (TIF) [file pone.0132987.s007.tif]

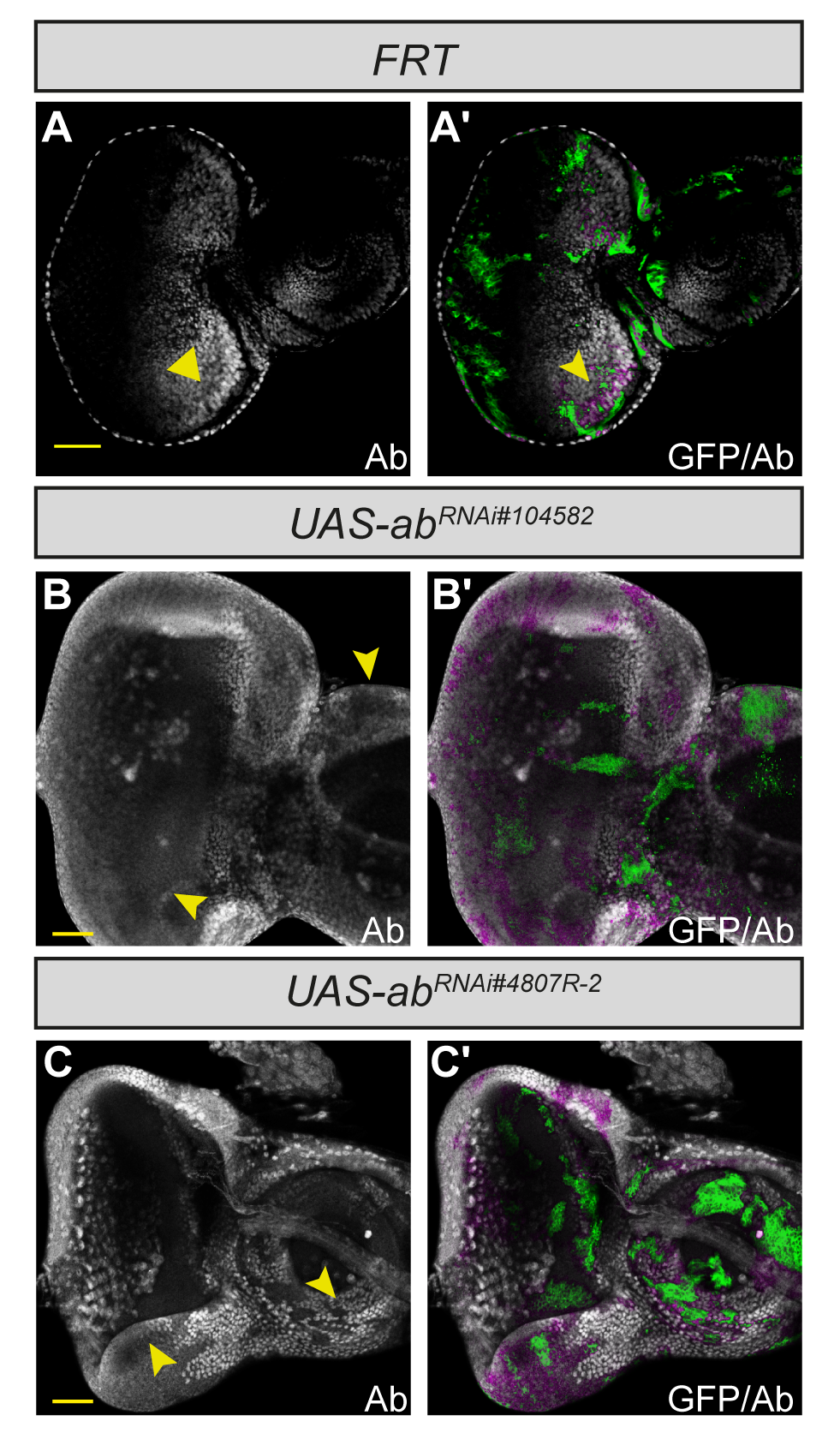

Supplement: S8 Fig — Mosaic eye-antennal discs, anterior to the right. Clones are generated with ey-FLP, and are positively marked by GFP (green, or magenta when overlaid with white). Ab is detected by immunohistochemical staining (white, or magenta when overlaid with GFP in the merges). Yellow scale bar corresponds to 40μM. (A-C) Control FRT82B mosaic discs show the endogenous expression of Ab in the eye progenitor domain (A, arrowhead) and antennal disc. UAS-ab RNAi#104582 expressing clones show decreased Ab protein levels (B, arrowheads). UAS-ab RNAi#4807R-2 expressing clones also show decreased Ab protein levels (C, arrowheads). (TIF) [file pone.0132987.s008.tif]

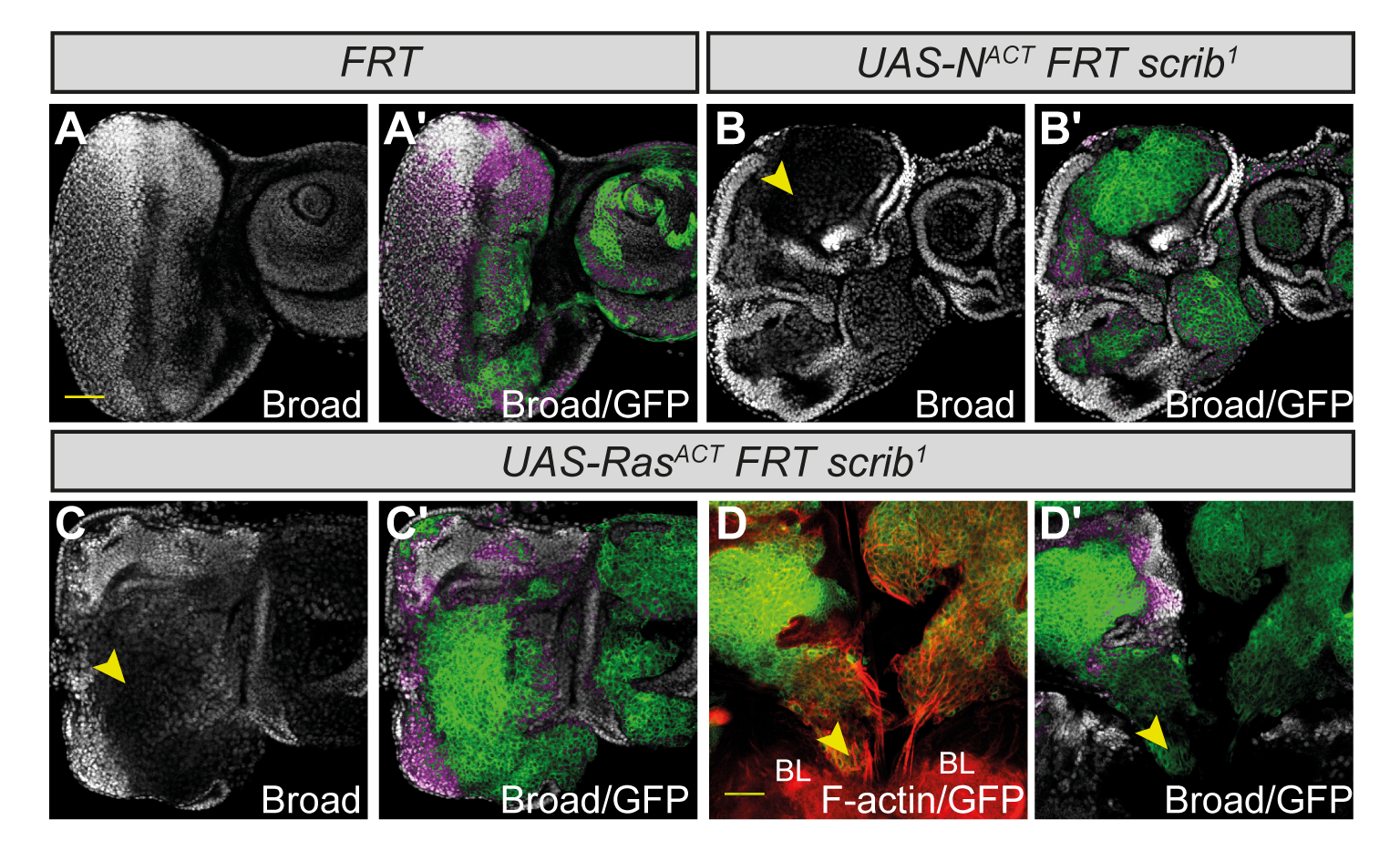

Supplement: S9 Fig — Mosaic eye-antennal discs, anterior to the right. Brain lobes (BL) are also shown in (D). Clones are generated with ey-FLP, and are positively marked by GFP (green, or magenta when overlaid with white). Br is detected by immunohistochemical staining (white, or magenta when overlaid with GFP in the merges). Tissue morphology is shown with phalloidin staining F-actin in (D, red). Yellow scale bar corresponds to 40μM. (A-D) In control FRT82B mosaic discs, Broad is expressed in both the eye and antennal disc (A). In scrib 1 + N ACT (B, arrowhead) and scrib 1 + RasACT tumors (C, arrowhead), Br levels are reduced. scrib 1 + Ras ACT tumor cells, which are known to be active for JNK-pathway activity [16], migrate between the brain lobes and do not express Br (D). (TIF) [file pone.0132987.s009.tif]

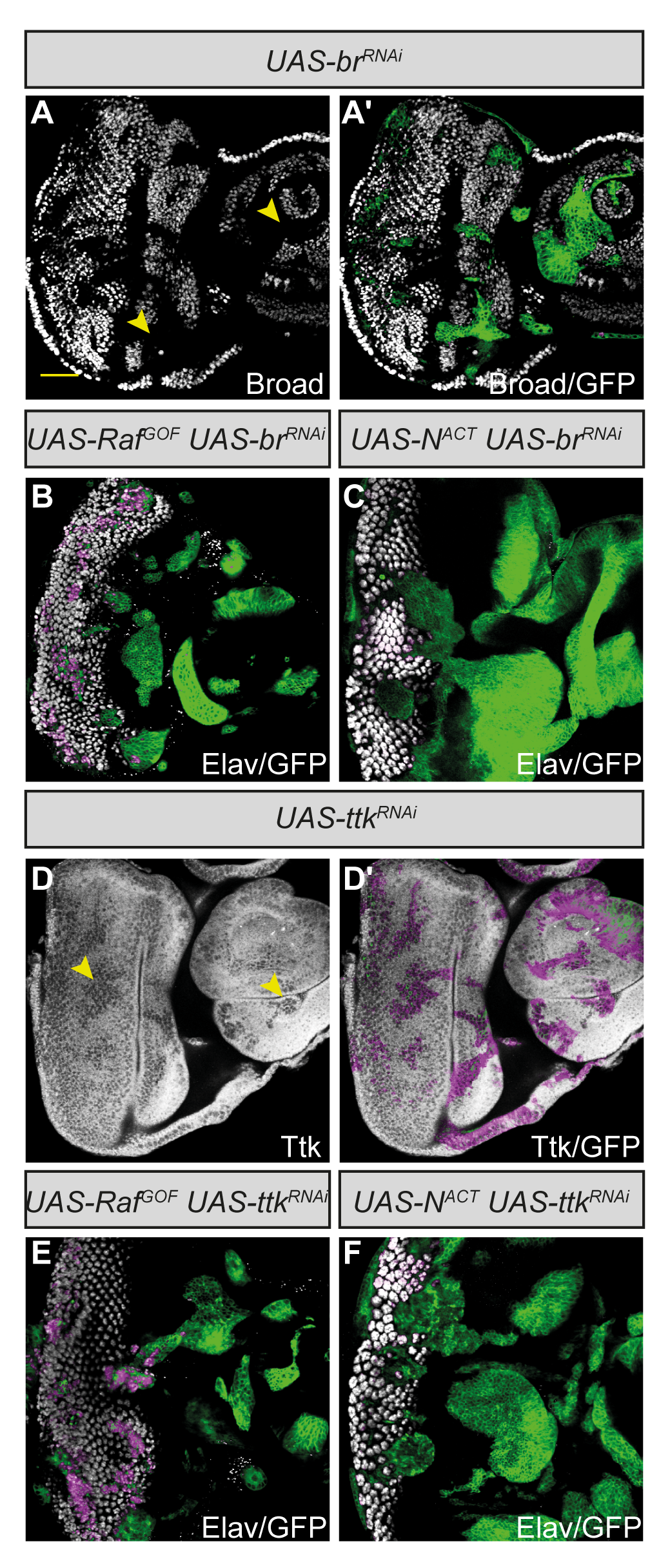

Supplement: S10 Fig — Mosaic eye-antennal discs, anterior to the right. Clones are generated with ey-FLP, and are positively marked by GFP (green, or magenta when overlaid with white). Br, Ttk and Elav are detected by immunohistochemical staining (white, or magenta when overlaid with GFP in the merges). Yellow scale bar corresponds to 40μM. (A-C) UAS-br RNAi#104648 expressing clones show decreased Br protein levels (A, arrowheads). Co-expressing UAS-br RNAi#104648 in UAS-Raf GOF (B) and UAS-N ACT (C) clones does not result in tumorigenesis. (D-F) Ttk levels are reduced in UAS-ttk RNAi#101980 expressing clones (D, arrowheads), and co-expression of UAS-ttk RNAi#101980 in UAS-Raf GOF (E) and UAS-N ACT (F) clones does not result in tumorigenesis. (TIF) [file pone.0132987.s010.tif]
