# Supplementary material for: BTB-Zinc Finger Oncogenes Are Required for Ras and Notch-Driven Tumorigenesis in Drosophila
Source: PLoS One. 2015 Jul 24;10(7):e0132987. doi: 10.1371/journal.pone.0132987 (PMC4514741; doi:10.1371/journal.pone.0132987)
Supplement: S1 Table — (DOC) [file pone.0132987.s013.doc]

**S1 Table.** Expression of candidate genes in *scrib^-^* + *Ras^ACT^* and *scrib^-^* + *N^ACT^* tumors (+/- *bsk^DN^*) compared to control *FRT82B* eye-antennal discs, and in *scrib^-^* + *Ras^ACT^* and *scrib^-^* + *N^ACT^* tumors compared to their respective genotypes expressing *bsk^DN^*.

| **Gene** | **Probe set** | **Log fold change in expression** | | | | | |
| --- | --- | --- | --- | --- | --- | --- | --- |
|  |  | **Compared to *FRT82B* control** | | | | **Compared to *bsk^DN^*** | |
|  |  | *scrib^-^* + *Ras^ACT^*  *vs*  *FRT82B* | *scrib^-^* + *Ras^ACT^* + *bsk^DN^*  *vs*  *FRT82B* | *scrib^-^* + *N^ACT^*  *vs*  *FRT82B* | *scrib^-^* + *N^ACT^* + *bsk^DN^*  *vs*  *FRT82B* | *scrib^-^* + *Ras^ACT^*  *vs*  *scrib^-^* + *Ras^ACT^* + *bsk^DN^* | *scrib^-^* + *N^ACT^*  *vs*  *scrib^-^* + *N^ACT^* + *bsk^DN^* |
| cher | 1632339_s_at | **1.524407** | n.s. | **2.383058** | **0.639905** | **1.697484** | **1.743153** |
| crb | 1628146_at | **-0.74716** | **-0.585494** | n.s. | n.s. | n.s. | n.s. |
| cycA | 1639195_a_at | n.s. | **-0.174243** | n.s. | **0.195694** | n.s. | **-0.238753** |
| cycD | 1627295_s_at | **-0.842681** | **-0.690502** | **-0.45774** | **-0.338267** | n.s. | n.s. |
| cycE | 1626249_s_at | n.s. | n.s. | n.s. | **0.710883** | **-0.523161** | **-0.705164** |
| dl | 1623415_at  1639692_s_at | **1.009793**  n.s. | **-1.268798**  **-0.561263** | n.s.  **0.447008** | **-1.240258**  n.s. | **2.278591**  **0.819785** | **1.3991**  **0.812698** |
| dm | 1625856_at | n.s. | n.s. | **0.726162** | n.s. | n.s. | **0.762995** |
| dpp | 1630026_s_at | n.s. | n.s. | n.s. | n.s. | n.s. | n.s. |
| E2f | 1625387_s_at | **-0.321408** | n.s. | n.s. | **-0.455351** | **-0.429585** | **0.236757** |
| E2f2 | 1627605_at | **-0.226362** | n.s. | **-0.334596** | **0.4431** | **-0.153002** | **-0.777696** |
| esg | 1641639_at | n.s. | n.s. | **0.540266** | **0.88379** | n.s. | n.s. |
| ex | 1625970_at | n.s. | n.s. | **0.577627** | **0.793707** | n.s. | n.s. |
| fra | 1623827_a_at | **-1.403273** | **-0.498188** | **-0.955819** | **-0.64243** | **-0.905086** | n.s. |
| fj | 1636091_at | **0.690285** | **-0.326728** | **0.729717** | n.s. | **1.017014** | **0.345944** |
| ft | 1624125_at | **-0.614412** | **-0.608975** | n.s. | n.s. | n.s. | n.s. |
| hpo | 1634303_at | **-0.399455** | **-0.311508** | **-0.379245** | **-0.329227** | n.s. | n.s. |
| Ilp8 | 1625664_at | **6.069047** | **2.659565** | **5.114234** | **3.57074** | **3.409482** | **1.543494** |
| Mef2 | 1626392_s_at  1628385_a_at  1626900_a_at  1636222_at | **0.741426**  **-0.555116**  n.s.  n.s. | n.s.  **-0.340571**  n.s.  n.s. | n.s.  n.s.  n.s.  n.s. | **-2.418151**  n.s.  **0.440251**  n.s. | **1.32656**  n.s.  n.s.  n.s. | **2.595394**  n.s  **-0.440251**  n.s |
| Mer | 1623379_at | n.s. | n.s. | n.s. | n.s. | n.s. | n.s. |
| Mmp1 | 1625761_a_at  1632204_at  1623160_at | **3.945943**  **3.043496**  3.149762 | n.s.  **-0.621023**  n.s. | **3.0913**  **1.779752**  **2.656003** | **-0.901045**  **-1.84099**  **-1.506674** | **4.235013**  **3.66452**  **3.254724** | **3.992344**  **3.620741**  **4.162677** |
| netA | 1624408_at | n.s. | n.s. | n.s. | n.s. | n.s. | n.s. |
| netB | 1636954_at | n.s. | **1.193711** | n.s. | n.s. | **-1.089692** | n.s. |
| Pax | 1626624_s_at | **2.068693** | **0.839044** | **1.629143** | n.s. | **1.229648** | **1.853047** |
| puc | 1631765_at | **0.869656** | **-0.647901** | **0.758023** | **-1.536673** | **1.517558** | **2.294696** |
| Pvf1 | 1631638_at | **1.567627** | **-1.271906** | **1.552201** | **-2.388859** | **2.839533** | **3.941061** |
| Pvf2 | 1630642_at | **0.681306** | n.s. | n.s. | **-2.197465** | **0.637253** | **2.415243** |
| Pvf3 | 1632817_at  1632773_a_at  1637519_at  1641276_at | n.s.  n.s.  n.s.  n.s. | n.s.  n.s.  n.s.  n.s. | n.s.  n.s.  n.s.  n.s. | n.s.  n.s.  n.s.  n.s. | n.s.  n.s.  n.s.  n.s. | n.s.  n.s.  n.s.  n.s. |
| Pvr | 1638143_a_at | n.s. | **-1.368582** | n.s. | **-2.022534** | **1.43962** | **2.044475** |
| rbf | 1623479_at | **-0.472871** | **-0.331761** | n.s. | **0.558039** | n.s. | **-0.770754** |
| Rbf2 | 1638806_at | n.s. | n.s. | n.s. | n.s. | n.s. | **-0.450727** |
| sav | 1628053_at | **-0.233154** | n.s. | n.s. | n.s. | **-0.178906** | n.s. |
| scaf | 1640912_s_at | **1.30752** | **0.604188** | **1.752096** | **-0.54302** | **0.703332** | **2.295116** |
| shg | 1639613_at | **-0.337955** | **-0.331266** | n.s. | n.s. | n.s. | n.s. |
| sna | 1636646_at | n.s. | n.s. | n.s. | n.s. | n.s. | n.s. |
| srp | 1629229_a_at | n.s. | **-2.021441** | n.s. | **-3.083344** | n.s. | **3.65491** |
| Stat92E | 1635827_s_at | n.s. | n.s. | n.s. | n.s. | n.s. | n.s. |
| th | 1636787_s_at | **-0.420782** | n.s. | n.s. | **0.647026** | **-0.469275** | **-0.745921** |
| twi | 1631682_at | **-0.11947** | **-0.11947** | **-0.11947** | **-0.11947** | n.s. | n.s. |
| upd-os | 1633968_at | **3.595729** | n.s. | **3.923185** | **3.046387** | **2.871169** | **0.876798** |
| upd2 | 1623718_at | **1.373645** | n.s. | **0.905782** | n.s. | **1.584487** | **1.025803** |
| upd3 | 1636892_at  1641119_at | **1.193158**  n.s. | n.s.  n.s. | **1.029579**  n.s. | n.s.  n.s. | **1.142148**  n.s. | **0.830614**  n.s. |
| wg | 1632868_a_at | **-0.802732** | n.s. | n.s. | **0.641161** | **-0.846983** | **-0.662093** |
| wor | 1631502_at | n.s. | n.s. | n.s. | n.s. | n.s. | n.s. |
| wts | 1635182_at | **-0.559422** | n.s. | n.s. | n.s. | n.s. | **-0.450866** |
| yki | 1628461_a_at | **-0.468319** | **-0.275255** | n.s. | n.s. | n.s. | **-0.232929** |
| Zfh1 | 1628262_a_at | n.s. | **-1.205177** | n.s. | **-3.090897** | n.s. | **2.973476** |
| Zfh2 | 1627320_at | **-1.402243** | **-1.03561** | **-1.130005** | **-1.493303** | n.s. | n.s. |

n.s. = not significant (p value>0.05)
