# Supplementary material for: BTB-Zinc Finger Oncogenes Are Required for Ras and Notch-Driven Tumorigenesis in Drosophila
Source: PLoS One. 2015 Jul 24;10(7):e0132987. doi: 10.1371/journal.pone.0132987 (PMC4514741; doi:10.1371/journal.pone.0132987)
Supplement: S2 Table — (DOC) [file pone.0132987.s014.doc]

**S2 Table.** Expression of BTB-ZF genes in *scrib^-^* + *Ras^ACT^* and *scrib^-^* + *N^ACT^* tumors (+/- *bsk^DN^*) compared to control *FRT82B* eye-antennal discs.

| **Gene** | **Probe set** | **Log fold change in expression compared to *FRT82B* control** | | | |
| --- | --- | --- | --- | --- | --- |
|  |  | *scrib^-^* + *Ras^ACT^* | *scrib^-^* + *Ras^ACT^* + *bsk^DN^* | *scrib^-^* + *N^ACT^* | *scrib^-^* + *N^ACT^* + *bsk^DN^* |
| ab (CG32830) | 1637140_at | n.s. | n.s. | n.s. | 0.33815 |
| br | 1636931_at  1629683_at  1637312_a_at  1626996_s_at  1635691_at | -3.061735  -2.445065  -2.648104  -2.838581  -2.029402 | -1.274922  -1.215787  -1.164036  -1.313666  -0.778866 | -1.262504  -1.370111  -2.356902  -1.751097  -1.235818 | 0.647421  n.s.  -0.783639  n.s.  -0.712193 |
| CG6765 | 1639157_at | n.s. | 2.164383 | -1.015132 | -2.160662 |
| CG6792 | 1639534_at | 0.688771 | 0.591199 | 0.443467 | 0.442852 |
| CG12236 | 1624189_at  1634451_at | -1.471121  n.s. | n.s.  n.s. | n.s.  n.s. | n.s.  n.s. |
| CG15725 | 1635339_at | n.s. | n.s. | n.s. | n.s. |
| CG32121 | 1628185_at | n.s. | n.s. | n.s. | n.s. |
| chinmo | 1629484_s_at  1628005_at  1636985_s_at | 2.664583  2.347146  n.s. | 0.87721  n.s.  n.s. | 3.576012  3.126264  n.s. | 1.230968  n.s.  n.s. |
| CP190 | 1631940_s_at | -0.693967 | n.s. | n.s. | n.s. |
| fru | 1629904_at  1632859_a_at  1634379_a_at  1631498_a_at  1624575_a_at  1641338_at  1638111_at | 1.663624  1.81059  2.013952  n.s.  n.s.  n.s.  n.s. | n.s.  n.s.  n.s.  n.s.  n.s.  n.s.  n.s. | 2.140687  2.258257  2.612835  1.848733  0.26459  3.058645  n.s. | n.s.  0.974944  1.477852  n.s.  n.s.  2.244114  n.s. |
| ken | 1628840_at | n.s. | n.s. | 0.750278 | n.s. |
| lola | 1633089_a_at  1624729_at  1640280_at  1634495_s_at  1630936_at  1633422_a_at  1627324_at  1637581_at  1625768_s_at 1628946_at  1635096_at  1641609_at  1629523_at  1640945_at  1628421_at  1623411_at | 0.894869  -1.9342  -0.592857  -0.489053  -1.07242  -0.532717  -1.246056  -1.20265  -0.95594  -1.059464  -1.154749  -1.22512  -1.252541  -1.149932  -1.366751  n.s. | n.s.  -1.310123  -0.561489  0.181452  -0.678811  n.s.  n.s.  n.s.  n.s.  n.s.  n.s.  n.s.  n.s.  n.s.  n.s.  0.391679 | 1.076851  -0.948939  n.s.  n.s.  -0.891305  n.s.  -0.937576  n.s.  -1.139199  n.s.  n.s.  n.s.  n.s.  -1.053243  n.s.  n.s. | 0.767757  -0.959451  n.s.  n.s.  -0.773885  0.260201  -1.352872  -0.990229  -1.555765  -0.868261  -1.047692  n.s.  n.s.  -1.888493  -1.510297  -0.164807 |
| mamo | 1638749_at  1632900_at | n.s.  n.s. | n.s.  n.s. | n.s.  n.s. | -1.490196  n.s. |
| Trl | 1628275_at  1629754_s_at  1635305_s_at | -0.971138  -0.439407  n.s. | n.s.  -0.318952  -0.544683 | -0.467674  n.s.  n.s. | n.s.  0.401191  0.415361 |
| ttk | 1637917_s_at  1636673_s_at | -1.769519  -1.108265 | -0.425144  n.s. | -1.290847  -0.568938 | n.s.  n.s. |

n.s. = not significant (p value>0.05)
